# Evolution of antithrombotic therapy for patients with atrial fibrillation: The prospective global GLORIA-AF registry program

**DOI:** 10.1371/journal.pone.0274237

**Published:** 2022-10-06

**Authors:** Lea Beier, Shihai Lu, Lionel Riou França, Sabrina Marler, Gregory Y. H. Lip, Menno V. Huisman, Christine Teutsch, Jonathan L. Halperin, Kristina Zint, Hans-Christoph Diener, Laurie Baker, Chang-Sheng Ma, Miney Paquette, Dorothee B. Bartels, Sergio J. Dubner, Philippe Lyrer, Jochen Senges, Kenneth J. Rothman

**Affiliations:** 1 Charité–Universitätsmedizin Berlin, Berlin, Germany; 2 Takeda Pharmaceuticals, Inc., Cambridge, Massachusetts, United States of America; 3 Sanofi-Aventis Recherche et Développement, Chilly-Mazarin, France; 4 Liverpool Centre for Cardiovascular Science, University of Liverpool and Liverpool Heart & Chest Hospital, Liverpool, United Kingdom; 5 Department of Clinical Medicine, Aalborg Thrombosis Research Unit, Aalborg University, Aalborg, Denmark; 6 Leiden University Medical Center, Leiden, the Netherlands; 7 Department of CardioMetabolism and Respiratory Medicine, Boehringer Ingelheim International GmbH, Ingelheim, Germany; 8 Icahn School of Medicine at Mount Sinai, New York City, New York, United States of America; 9 University Hospital Essen, Essen, Germany; 10 Boehringer Ingelheim, Burlington, Canada; 11 Atrial Fibrillation Center, Beijing Anzhen Hospital, Beijing, People’s Republic of China; 12 Hannover Medical School, Hannover, Germany; 13 UCB Biosciences GmbH, Monheim, Germany; 14 Clínica y Maternidad Suizo Argentina, Buenos Aires, Argentina; 15 University Hospital Basel, Stroke Center Neurology, and University of Basel, Basel, Switzerland; 16 Stiftung Institut, Ludwigshafen, Germany; 17 RTI Health Solutions, Research Triangle Institute, Research Triangle Park, North Carolina, United States of America; University of Messina, ITALY

## Abstract

**Objective:**

To assess baseline characteristics and antithrombotic treatment (ATT) prescription patterns in patients enrolled in the third phase of the GLORIA-AF Registry Program, evaluate predictors of treatment prescription, and compare results with phase II.

**Methods:**

GLORIA-AF is a large, global, prospective registry program, enrolling patients with newly diagnosed nonvalvular atrial fibrillation (AF) at risk of stroke. Patients receiving dabigatran were followed for two years in phase II, and all patients were followed for 3 years in phase III. Phase II started when dabigatran became available; phase III started when the characteristics of patients receiving dabigatran became roughly comparable with those receiving vitamin K antagonists (VKAs).

**Results:**

Between 2014 and 2016, 21,241 patients were enrolled in phase III. In total, 82% of patients were prescribed oral anticoagulation ([OAC]; 59.5% novel/nonvitamin K oral anticoagulants [NOACs], 22.7% VKAs). A further 11% of patients were prescribed antiplatelets without OAC and 7% were prescribed no ATT. A high stroke risk was the main driver of OAC prescription. Factors associated with prescription of VKA over NOAC included type of site, region, physician specialty, and impaired kidney function.

**Conclusion:**

Over the past few years, data from phase III of GLORIA-AF show that OACs have become the standard treatment option, with most newly diagnosed AF patients prescribed a NOAC. However, in some regions a remarkable proportion of patients remain undertreated. In comparison with phase II, more patients received NOACs in phase III while the prescription of VKA decreased. VKAs were preferred over NOACs in patients with impaired kidney function.

## Introduction

With a lifetime risk of up to 26%, atrial fibrillation (AF) is the most common cardiac arrythmia [1]. AF is an important contributor to population mortality and morbidity [2, 3] and an independent risk factor for stroke [4]. The stroke risk can be reduced by two-thirds with antithrombotic treatment (ATT) [5, 6]. Multiple options for ATT with differing effectiveness and safety profiles are available.

The introduction of novel/nonvitamin K oral anticoagulants (NOACs) expanded the choices for stroke prevention in nonvalvular AF patients and is reflected in major changes in updated versions of guidelines on the management of AF. Guideline-adherent ATT is associated with better clinical outcomes, emphasizing the importance of implementation and adherence to guidelines [7–9].

The Global Registry on Long-Term Oral Antithrombotic Treatment in Patients with Atrial Fibrillation (GLORIA-AF, NCT01468701) was a large, global, prospective observational registry program, that enrolled patients with newly diagnosed nonvalvular AF at risk of stroke. In earlier phases of the registry program, substantial regional differences in prescription patterns of ATT were found [10], and a large percentage of patients worldwide were undertreated [11]. The objective of the present report was to describe patient characteristics and ATT patterns in the last phase of the GLORIA-AF registry program (phase III) and evaluate predictors of treatment prescription as well as compare results with those from phase II.

## Methods

The design of GLORIA-AF was reported previously [12]. The program had 3 phases: phase I was conducted before approval of NOACs (at the local level, and enrolled patients between May 2011 and January 2013); phase II started after approval of dabigatran (at the local level, and enrolled patients between November 2011 and December 2014); phase III began when baseline characteristics of patients receiving dabigatran treatment became similar to that of patients receiving vitamin K antagonist (VKA) treatment based on data from phase II (and enrolled patients between January 2014 and December 2016) [12]. In phase II, patients receiving dabigatran were followed for 2 years and in phase III all patients were followed for 3 years, irrespective of ATT status. Patients were eligible for enrollment in the GLORIA-AF registry program if they were aged ≥18 years (Japan ≥20 years), newly diagnosed (<3 months before the baseline visit; Latin America <4.5 months) with nonvalvular AF, and at risk of stroke (CHA_2_DS_2_-VASc [congestive heart failure, hypertension, age ≥75 years, diabetes, stroke/transient ischemic attack/systemic embolism, vascular disease, age from 65–74 years, sex category (female)] score ≥1) [12]. Patients were classified into four groups according to their prescribed ATT: NOACs, VKAs, antiplatelets (APs) without oral anticoagulation (OACs) (alone), no ATTs. Additionally, these groups were grouped into OAC (i.e., NOAC or VKA) or no OAC (i.e., APs or no ATT). Patients receiving combination therapy with an OAC and AP were classified into the specific OAC group (i.e., NOAC or VKA). To increase comparability of the study phases, patients from Africa/the Middle East were excluded from all analyses as they were only enrolled in phase II. The study was conducted in accordance with the Declaration of Helsinki. The protocol was approved by the European Medicines Agency and institutional review boards at each participating site. An independent, academic steering committee oversaw the design, execution, study conduct, and publication planning. Members of the steering committee meeting International Committee of Medical Journal Editors authorship criteria were responsible for manuscript development.

Collection of data in the study was managed during routine practice visits (or via telephone in exceptional circumstances). Clinical data and site characteristics were captured using a secure web-based electronic data capture system which ensured confidentiality and data integrity.

### Statistical analyses

Categorical variables were summarized by absolute frequencies and percentages; continuous variables were summarized by mean and standard deviation. Descriptive analyses were performed for all patients enrolled in phase III and stratified by treatment group (i.e., NOAC vs. VKA vs. AP vs. no ATT). Baseline data from patients enrolled in phase II were analyzed and compared with baseline characteristics from phase III. Standardized differences were used to assess comparability; differences ≤0.1 were considered to reflect reasonable balance [13].

Factors associated with ATT choice in phase III were analyzed by log-binomial multivariable regression models, and relative probability ratios were provided for prescription (i.e., OAC vs. no OAC, NOAC vs. VKA, AP vs. no ATT). Two models were examined for each analysis to allow for the evaluation of the impact of risk scores (i.e., CHA_2_DS_2_-VASc [low = 1 in female, moderate = 1 in male or 2 in female, high ≥2 in male or ≥3 in female] and HAS-BLED [hypertension, abnormal renal/liver function, stroke, bleeding history or predisposition, labile international normalized ratio, elderly (>65 years), drugs or alcohol concomitantly; low <3, high ≥3]) that are recommended in treatment decision guidelines, and the impact of individual score components while avoiding multicollinearity. One model included all variables of interest and the risk scores (Model 1) and the other model included all variables of interest and the components of the risk score indices, but not the scores themselves (Model 2). Since including the individual components rather than the scores should lead to better predictive performance and help identify if some components were more strongly associated than others with prescription decisions, we herein present results from the model based on the risk score components (Model 2); see supplement S1 File for Model 1 results.

The COPY method was used to obtain approximate maximum likelihood estimates when the log-binomial model did not converge [14]. Missing data were handled using multiple imputation, which imputed missing data using multiple, independently simulated values based on models to provide comparatively unbiased estimates of missing values under the missing-at-random assumption, with added random error to compensate for the imputed information [15]. Each imputed dataset was analyzed separately and the results were combined to provide the final estimates.

Marginal fitted probabilities of all treatment groups (i.e., OAC vs. no OAC, NOAC vs. VKA, AP vs. no ATT) in both phases, and their differences, were computed by logistic regression using a combination of the phase II and phase III cohorts. With the exception of total risk scores, the covariates included in the logistic regression were the same as those in the log-binomial regression. The 95% confidence intervals (CIs) were estimated using a bootstrap approach. For each of the 20 imputed datasets, 200 bootstrap samples (also called bootstrap replicates) were drawn, which yielded 20*200 (4000) datasets D_m,b_; m = 1, …, 20; b = 1, …, 200. In each of these datasets the quantity of interest was estimated and then the resultant 4000 estimates were used to construct the desired CIs by percentile method [16].

A cluster analysis was used to examine the treatment pattern of the phase III patients by country. The k-means algorithm approach was applied to prescription levels and the number of clusters was determined by the simple rule of thumb: $k\approx \sqrt{n/2}$ with *n* as the number of countries [17].

All analyses were performed using SAS software version 9.4 (SAS Institute, Inc., Cary, NC, USA).

## Results

### Baseline characteristics

Baseline characteristics of patients enrolled in phase III by ATT are presented in Table 1; additional characteristics are presented in S1 Table in S1 File. Between 2014 and 2016, 21,241 eligible patients were enrolled in phase III of GLORIA-AF in 931 sites from 38 countries. Most patients received OACs (82.2%): 59.5% received NOACs (apixaban 21.1%, rivaroxaban 18.9%, dabigatran 18.0%, edoxaban 1.6%), and 22.7% received VKAs. Additionally, 11.2% of the patients were prescribed APs without OAC and 6.6% received no ATTs.

**Table 1:** Baseline characteristics by ATT for phase III patients enrolled between 2014 and 2016.

N (%) or mean ± SD	All	NOAC (± AP)	VKA (± AP)	AP	No ATT
	N = 21,241	N = 12,637	N = 4828	N = 2373	N = 1403
Patient characteristics					
Age (years)	70.5 ± 10.6	71.0 ± 10.2	71.1 ± 10.4	68.5 ± 11.8	67.5 ± 12.2
Sex, female	9546 (44.9)	5704 (45.1)	2147 (44.5)	1027 (43.3)	668 (47.6)
BMI (kg/m^2^)*	28.6 ± 6.4	29.0 ± 6.6	28.6 ± 6.1	27.3 ± 5.8	26.9 ± 5.8
Medical history					
Hypertension^†^					
Uncontrolled	2157 (10.2)	1097 (8.7)	470 (9.7)	409 (17.2)	181 (12.9)
Controlled	13,358 (62.9)	8362 (66.2)	3090 (64.0)	1229 (51.8)	677 (48.3)
Hyperlipidemia	8296 (39.1)	5273 (41.7)	1844 (38.2)	841 (35.4)	338 (24.1)
Diabetes mellitus	4940 (23.3)	2931 (23.2)	1229 (25.5)	531 (22.4)	249 (17.7)
Congestive heart failure	4616 (21.7)	2480 (19.6)	1381 (28.6)	516 (21.7)	239 (17.0)
Coronary artery disease	3967 (18.7)	2129 (16.8)	921 (19.1)	763 (32.2)	154 (11.0)
Stroke/transient ischemic attack/systemic embolism	3086 (14.5)	1893 (15.0)	648 (13.4)	363 (15.3)	182 (13.0)
Vascular disease^‡^	2691 (12.7)	1430 (11.3)	712 (14.7)	462 (19.5)	87 (6.2)
Cancer	2112 (9.9)	1318 (10.4)	478 (9.9)	182 (7.7)	134 (9.6)
Chronic gastrointestinal diseases	2814 (13.2)	1740 (13.8)	564 (11.7)	319 (13.4)	191 (13.6)
Transient ischemic attack	948 (4.5)	638 (5.0)	198 (4.1)	73 (3.1)	39 (2.8)
Abnormal kidney function^§^	389 (1.8)	107 (0.8)	171 (3.5)	82 (3.5)	29 (2.1)
AP drug use^||^	5425 (25.5)	2165 (17.1)	888 (18.4)	2270 (95.7)	102 (7.3)
Interventions in AF					
Cardioversion	3840 (18.1)	2495 (19.7)	690 (14.3)	427 (18.0)	228 (16.3)
AF ablation	382 (1.8)	254 (2.0)	84 (1.7)	23 (1.0)	21 (1.5)
Region					
Asia	4239 (20.0)	1811 (14.3)	798 (16.5)	1032 (43.5)	598 (42.6)
Europe	10,279 (48.4)	6435 (50.9)	2747 (56.9)	587 (24.7)	510 (36.4)
North America	5097 (24.0)	3527 (27.9)	734 (15.2)	619 (26.1)	217 (15.5)
Latin America	1626 (7.7)	864 (6.8)	549 (11.4)	135 (5.7)	78 (5.6)
CHA_2_DS_2-_VASc score					
Low (1 for women)	488 (2.3)	168 (1.3)	73 (1.5)	118 (5.0)	129 (9.2)
Moderate (1 for men or 2 for women)	3967 (18.7)	2229 (17.6)	759 (15.7)	594 (25.0)	385 (27.4)
High (≥2 for men or ≥3 for women)	16,786 (79.0)	10,240 (81.0)	3996 (82.8)	1661 (70.0)	889 (63.4)
HAS-BLED score^¶^					
Low (<3)	17,242 (81.2)	10,517 (83.2)	3945 (81.7)	1598 (67.3)	1182 (84.2)
High (≥3)	1970 (9.3)	909 (7.2)	370 (7.7)	598 (25.2)	93 (6.6)
Type of AF					
Paroxysmal	11,972 (56.4)	7140 (56.5)	2179 (45.1)	1716 (72.3)	937 (66.8)
Persistent	7249 (34.1)	4333 (34.3)	1968 (40.8)	553 (23.3)	395 (28.2)
Permanent	2020 (9.5)	1164 (9.2)	681 (14.1)	104 (4.4)	71 (5.1)
Categorization of AF					
Symptomatic	6588 (31.0)	3794 (30.0)	1611 (33.4)	745 (31.4)	438 (31.2)
Minimally symptomatic	7216 (34.0)	4185 (33.1)	1638 (33.9)	865 (36.5)	528 (37.6)
Asymptomatic	7437 (35.0)	4658 (36.9)	1579 (32.7)	763 (32.2)	437 (31.1)
Physician specialty					
GP/PCP/geriatrician	1055 (5.0)	574 (4.5)	225 (4.7)	140 (5.9)	116 (8.3)
Cardiologist	18,056 (85.0)	10,840 (85.8)	3939 (81.6)	2108 (88.8)	1169 (83.3)
Neurologist	524 (2.5)	383 (3.0)	70 (1.4)	37 (1.6)	34 (2.4)
Internist	820 (3.9)	414 (3.3)	324 (6.7)	49 (2.1)	33 (2.4)
Other	779 (3.7)	422 (3.3)	267 (5.5)	39 (1.6)	51 (3.6)
Type of site					
GP/primary care	1318 (6.2)	683 (5.4)	254 (5.3)	248 (10.5)	133 (9.5)
Specialist office	6216 (29.3)	4090 (32.4)	1107 (22.9)	719 (30.3)	300 (21.4)
Community hospital	6252 (29.4)	4167 (33.0)	1243 (25.7)	502 (21.2)	340 (24.2)
University hospital	6756 (31.8)	3402 (26.9)	1947 (40.3)	804 (33.9)	603 (43.0)
Outpatient healthcare center	335 (1.6)	100 (0.8)	163 (3.4)	64 (2.7)	8 (0.6)
Anticoagulation clinics	118 (0.6)	45 (0.4)	57 (1.2)	8 (0.3)	8 (0.6)
Other	246 (1.2)	150 (1.2)	57 (1.2)	28 (1.2)	11 (0.8)

AF, atrial fibrillation; AP, antiplatelets; ATT, antithrombotic treatment; BMI, body mass index; CHA_2_DS_2_-VASc, congestive heart failure, hypertension, age ≥75 years, diabetes, stroke/transient ischemic attack/systemic embolism, vascular disease, age from 65–74 years, sex category (female); GP, general practitioner; HAS-BLED, hypertension, abnormal renal/liver function, stroke, bleeding history or predisposition, labile international normalized ratio, elderly (>65 years), drugs or alcohol concomitantly; PCP, primary care physician; NOAC, novel/nonvitamin K oral anticoagulants; SD, standard deviation; VKA, vitamin K antagonist.

*Data missing for 245 (1.2%) overall patients.

^†^Uncontrolled hypertension was defined as a history of hypertension and a current uncontrolled systolic blood pressure >160mmHg. Controlled hypertension was defined as a history of hypertension and a current uncontrolled systolic blood pressure ≤160mmHg.

^‡^Defined as prior myocardial infarction, peripheral artery disease, complex aortic plaque.

^§^Defined as presence of chronic dialysis or renal transplantation or serum creatinine ≥200 μmol/L.

^||^The AP treatment group includes patients *prescribed* ATT. AP use includes patients who *used* AP at the baseline visit.

^¶^Data missing for 2029 (9.6%) overall patients.

In the overall patient population, hypertension (73.1%) was the most prevalent comorbidity, followed by hyperlipidemia (39.1%) and diabetes mellitus (23.3%).

Baseline characteristics by study phase are presented in S2 Table in S1 File. Overall, the study cohorts of both phases were similar.

#### Marginal fitted probability of treatment choice

The marginal fitted probabilities of treatment choice are presented in Table 2. The fitted probability of receiving OAC treatment was 80.2% (95% CI, 80.0–80.4) in phase II and 82.0% (95% CI, 81.8–82.2) in phase III. The relative proportion of VKA over NOAC treatment among patients prescribed OACs decreased from 40.4% (95% CI, 40.2–40.6) in phase II to 27.9% (95% CI, 27.8–28.1) in phase III. The absolute probability difference for OAC treatment between both phases was 1.8% (95% CI,1.8–1.9)

**Table 2:** Multivariable logistic regression model (Model 2)* estimates of fitted probabilities of ATT prescription.

ATT strategy	Marginal fitted probability		
	(95% CIs)^†^		
	Phase II^‡^	Phase III	Difference
OAC ± AP^§^	0.802	0.820	0.018 (0.018, 0.019)
NOAC ± AP	0.596	0.721	0.125 (0.123, 0.126)
VKA ± AP	0.404	0.279	–0.125 (–0.126, –0.123)
No OAC^§^	0.198	0.180	–0.018 (–0.019, –0.018)
AP alone	0.622	0.619	–0.003 (–0.005, –0.001)
No ATT	0.378	0.381	0.003 (0.001, 0.005)

AP, antiplatelets; ATT, antithrombotic treatment; CI, confidence interval; NOAC, novel/nonvitamin K oral anticoagulants; OAC, oral anticoagulation; VKA, vitamin K antagonist.

*Model 2 considers body mass index, components of CHA_2_DS_2_−VASc (congestive heart failure, hypertension, age ≥75 years, diabetes, stroke/transient ischemic attack/systemic embolism, vascular disease, age from 65–74 years, sex category [female]) score, categorized components of HAS-BLED (hypertension, abnormal renal/liver function, stroke, bleeding history or predisposition, labile international normalized ratio, elderly [>65 years], drugs or alcohol concomitantly) score, region, and other variables (i.e., type of AF, chronic kidney disease, cancer, hyperlipidemia, coronary artery disease, smoking status, antiplatelet drug use, physician specialty, chronic gastrointestinal diseases, medical treatment reimbursed by, and type of site) for the analysis.

^**†**^Confidence intervals were constructed using the bootstrap method.

^‡^Excluding patients from Africa/the Middle East.

^§^AP use was defined as use at the baseline visit.

### Predictors of ATT choice

#### OAC versus no OAC

**Fig 1:**
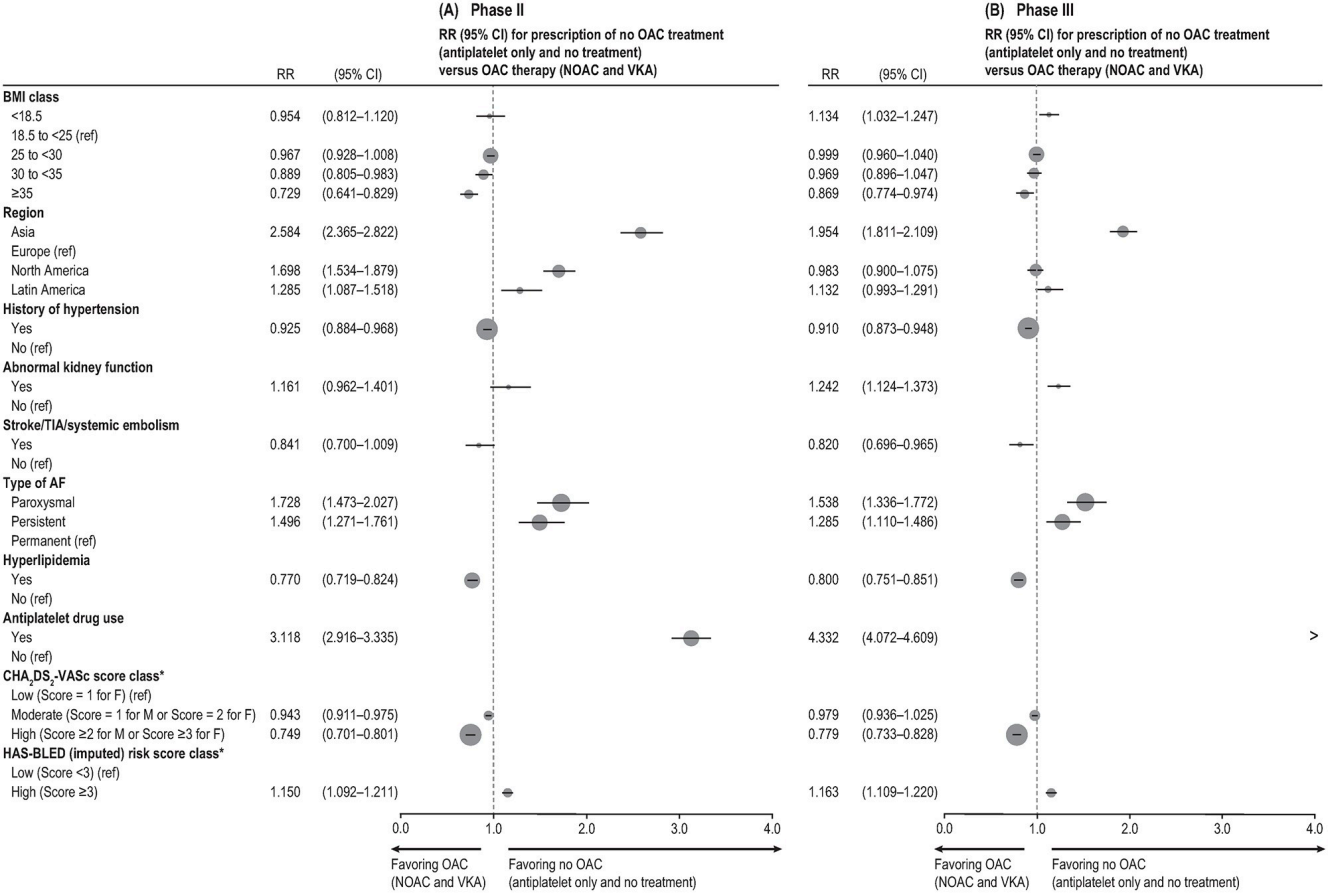
Factors most strongly associated with prescription of no OAC treatment (AP only and no treatment) versus OAC therapy (NOAC and VKA) in phase II (A) and phase III (B). AF, atrial fibrillation; BMI, body mass index; CHA_2_DS_2_−VASc (congestive heart failure, hypertension, age ≥75 years, diabetes, stroke/transient ischemic attack/systemic embolism, vascular disease, age from 65–74 years, sex category [female]); CI, confidence interval; F, female; HAS-BLED (hypertension, abnormal renal/liver function, stroke, bleeding history or predisposition, labile international normalized ratio, elderly [>65 years], drugs or alcohol concomitantly); M, male; NOAC, novel/nonvitamin K oral anticoagulants; OAC, oral anticoagulation; ref, reference; RR, relative risk; TIA, transient ischemic attack; VKA, vitamin K antagonist. *Effect estimates obtained from the model including all variables of interest and the risk scores, but not the components of the risk scores.

Factors most strongly associated with the prescription of no OAC (i.e., AP alone or no ATT) versus OAC therapy (i.e., NOAC or VKA) in phase III are presented in Fig 1 (Model 2); see **S3** and S4 Tables in S1 File for all factors assessed in Models 1 and 2.

The factor most strongly associated with no OAC was baseline AP drug use (marginal fitted probability ratio: 4.33; 95% CI, 4.07–4.61).

The strongest predictor of OAC prescription versus no OAC was a high stroke risk score (CHA_2_DS_2_-VASc ≥2 for male patients or ≥3 for female patients) versus a low risk score (CHA_2_DS_2_-VASc = 1 in female patients) (0.78; 95% CI, 0.73–0.83).

#### NOAC versus VKA

**Fig 2:**
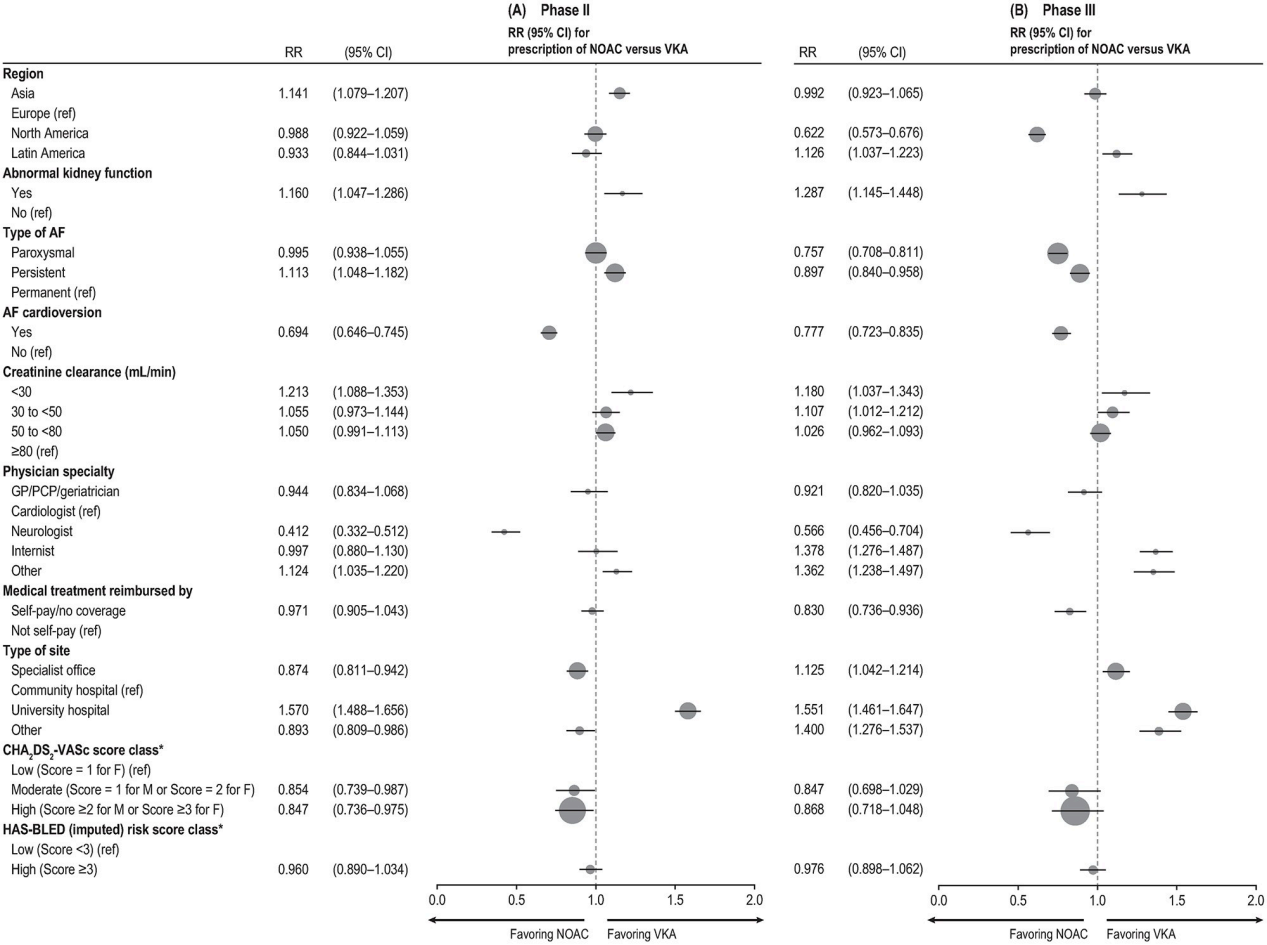
Factors most strongly associated with prescription of NOAC versus VKA in phase II (A) and phase III (B). AF, atrial fibrillation; CHA_2_DS_2_−VASc (congestive heart failure, hypertension, age ≥75 years, diabetes, stroke/transient ischemic attack/systemic embolism, vascular disease, age from 65–74 years, sex category [female]); CI, confidence interval; F, female; GP, general practitioner; HAS-BLED (hypertension, abnormal renal/liver function, stroke, bleeding history or predisposition, labile international normalized ratio, elderly [>65 years], drugs or alcohol concomitantly); M, male; NOAC, novel/nonvitamin K oral anticoagulants; PCP, primary care physician; ref, reference; RR, relative risk; TIA, transient ischemic attack; VKA, vitamin K antagonist. *Effect estimates obtained from the model including all variables of interest and the risk scores, but not the components of the risk scores.

Factors most strongly associated with prescription of VKA versus NOAC treatment in patients treated with OACs in phase III are reported in Fig 2 (Model 2); see S5 and S6 Tables in S1 File for all factors assessed in Models 1 and 2.

The strongest predictor of VKA prescription was enrollment in a university hospital versus a community hospital (1.55; 95% CI, 1.46–1.65).

The factors most strongly associated with prescription of NOAC versus VKA included enrollment by a neurologist versus a cardiologist (0.57; 95% CI, 0.46–0.70) and enrollment in North America versus Europe (0.62; 95% CI, 0.57–0.68).

#### AP versus no ATT

**Fig 3:**
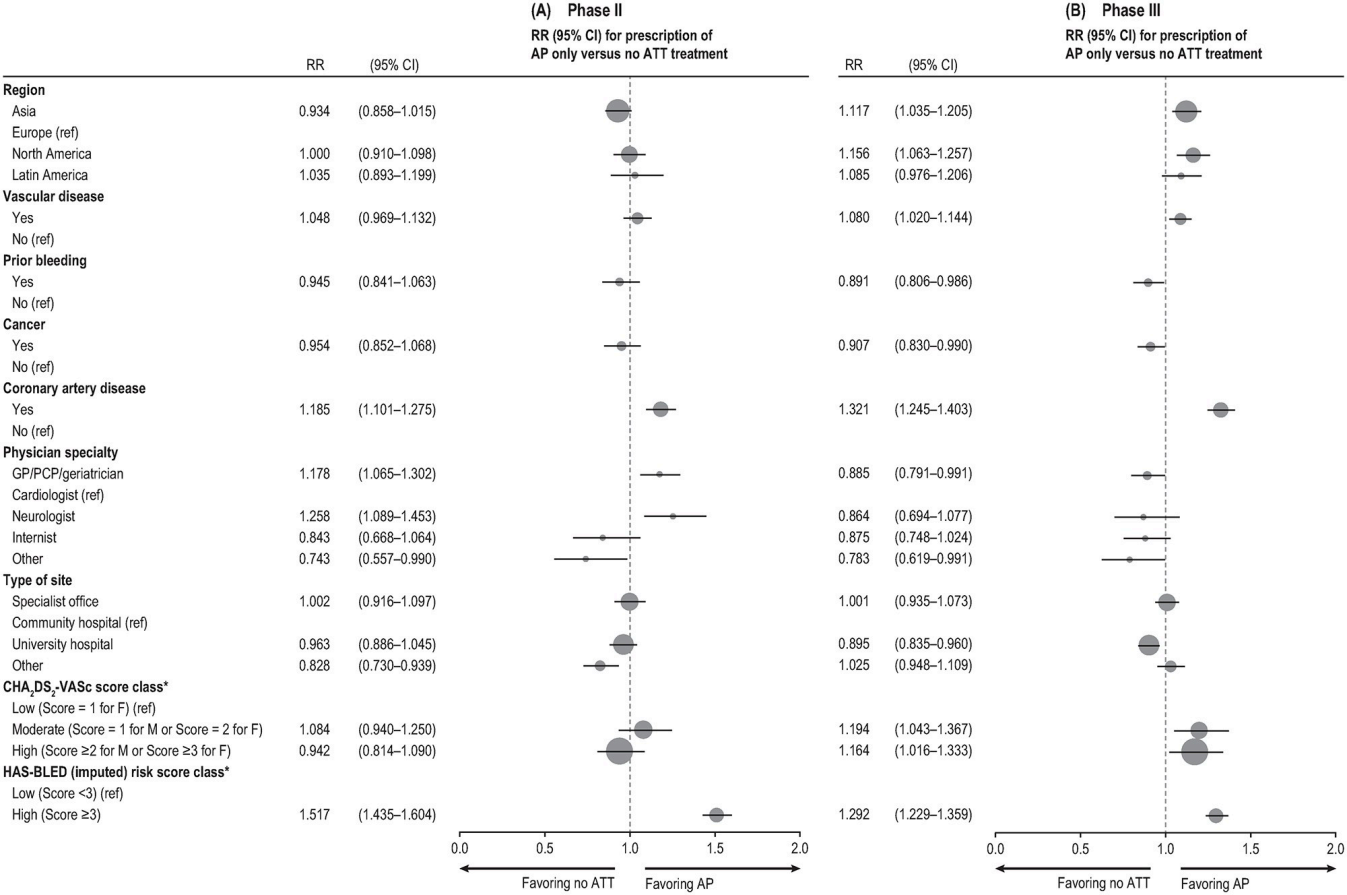
Factors most strongly associated with prescription of AP only versus no ATT in phase II (A) and phase III (B). AP, antiplatelet; ATT, antithrombotic treatment; CHA_2_DS_2_−VASc (congestive heart failure, hypertension, age ≥75 years, diabetes, stroke/transient ischemic attack/systemic embolism, vascular disease, age from 65–74 years, sex category [female]); CI, confidence interval; F, female; GP, general practitioner; HAS-BLED (hypertension, abnormal renal/liver function, stroke, bleeding history or predisposition, labile international normalized ratio, elderly [>65 years], drugs or alcohol concomitantly); M, male; PCP, primary care physician; ref, reference; RR, relative risk. *Effect estimates obtained from the model including all variables of interest and the risk scores, but not the components of the risk scores.

Results of the regression analysis for factors most strongly associated with prescription of AP versus no ATT are reported in Fig 3 (Model 2); see S7 and S8 Tables in S1 File for all factors assessed in Models 1 and 2.

In phase III, the factor most strongly associated with prescription of AP compared with no treatment was coronary artery disease (1.32; 95%C, 1.25–1.40), followed by a high bleeding risk (HAS-BLED score ≥3 vs. <3: 1.29; 95% CI, 1.23–1.36).

The factors most strongly associated with no ATT compared with AP were physician specialty (other specialty vs. cardiologist 0.78; 95% CI, 0.62–0.99, and general practitioner/primary care physician/geriatrician vs. cardiologist 0.89; 95% CI, 0.79–0.99).

### Treatment patterns by country

**Fig 4:**
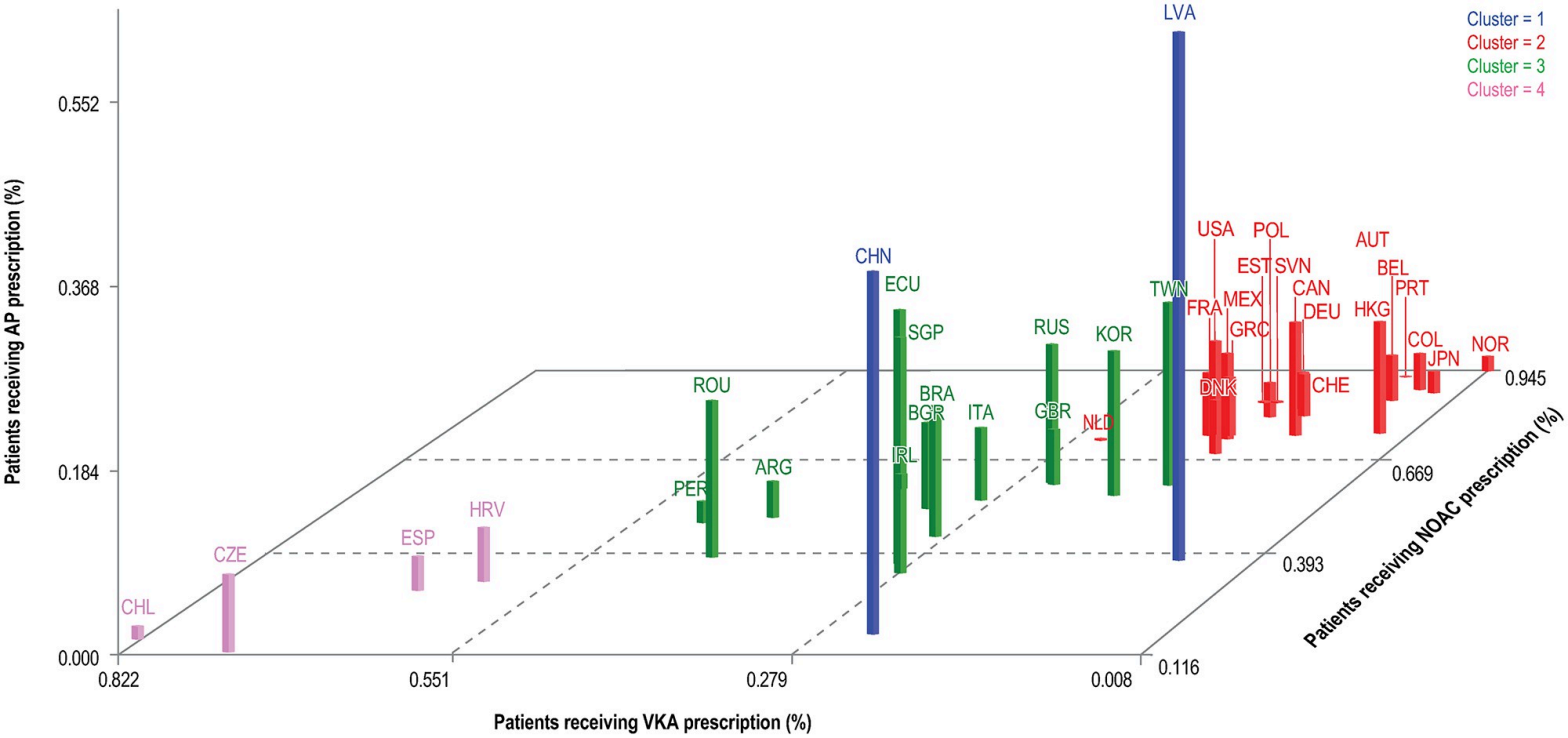
Analysis of ATT pattern in phase III by countries. AP, antiplatelet; ARG, Argentina; ATT, antithrombotic treatment; AUT, Austria; BEL, Belgium; BGR, Bulgaria; BRA, Brazil; CAN, Canada; CHE, Switzerland; CHL, Chile; CHN, China; COL, Colombia; CZE, Czech Republic; DNK, Denmark; DEU, Germany; ECU, Ecuador; ESP, Spain; EST, Estonia; FRA, France; GBR, United Kingdom of Great Britain and Northern Ireland; GRC, Greece; HKG, Hong Kong; HRV, Croatia; IRL, Ireland; ITA, Italy; JPN, Japan; KOR, Korea; LVA, Latvia; MEX, Mexico; NLD, the Netherlands; NOAC, novel/nonvitamin K oral anticoagulants; NOR, Norway; PER, Peru; POL, Poland; PRT, Portugal; ROU, Romania; RUS, Russian Federation; SGP, Singapore; SVN, Slovenia; TWN, Taiwan; USA, United States of America; VKA, vitamin K antagonist.

Results of the clustering analysis for the ATT pattern in phase III by countries with the number of clusters set as 4 ($\approx \sqrt{38/2}$) are presented in Fig 4.

Cluster 1, consisting of countries with a high prescription of AP only, included Latvia (55.2%) and China (37.4%). Most of the participating countries were assigned to Cluster 2, consisting of countries with a NOAC prescription in more than two-thirds of the patients. The highest proportions of NOAC prescriptions were found in Norway (94.5%), Portugal (92.0%), and Austria (91.1%) followed by Colombia (88.1%). Cluster 3, with intermediate NOAC (i.e., one- to two-thirds of the patients), AP, and VKA prescription, included most of the countries from Latin America, and all the remaining participating Asian countries, as well as 5 European countries (the United Kingdom, Italy, Bulgaria, Romania, and Ireland). Cluster 4, consisting of countries with high prescription of VKA with less than one-third of the patients receiving a NOAC, included Chile (VKA, 82.2%; NOAC, 14.9%), Spain (VKA, 65.1%; NOAC, 28.7%), Croatia (VKA, 60.7%; NOAC, 31.4%), and the Czech Republic (VKA, 73.3%; NOAC, 11.6%).

## Discussion

Data from phase III of the GLORIA-AF Registry Program showed that NOAC prescription has become more common for patients with newly diagnosed nonvalvular AF at risk of stroke. Compared with phase II results, among patients prescribed with OACs, the fitted marginal probability of receiving a NOAC increased by 12.5 percentage points in phase III (phase II, 59.6%; phase, III 72.1%) whereas the probability of receiving a VKA decreased (phase II, 40.4%; phase III, 27.9%). These findings indicate a widespread uptake of NOACs, which reflects the recommendations of current guidelines: The European Guideline for the Management of AF states that NOAC treatment is preferred over VKA treatment in patients eligible for OAC treatment [6]. Whilst the 2014 American Heart Association (AHA)/American College of Cardiology (ACC)/Heart Rhythm Society (HRS) Guideline did not give a recommendation as to which of the OACs should be preferred in general [18], the 2019 version of the AHA/ACC/HRS Guideline was updated, stating that NOACs are recommended over VKA in AF-patients eligible for NOAC treatment [19]. The increased use of NOACs is in line with findings from previous registries, showing an increase in NOAC prescription compared to VKA over the last years [20, 21].

However, among the patients who were not prescribed an OAC, the use of APs and no ATT did not change much over the years, with only a 0.3% difference of the fitted marginal probabilities for the prescription of APs alone versus no antithrombotic prescription between phase II and phase III on a global level. While, in contrast to our findings, a decrease in treatment with APs alone since the introduction of NOACs was previously shown, the finding that the proportion of patients not receiving any OAC did not decline over time was reported previously [21]. The country composition was similar between both phase II and phase III of the GLORIA-AF Registry Program. Among the patients treated with APs alone or without ATT, we found a high proportion of patients with a high risk for stroke (70.0% and 63.4%, respectively), indicating that a non-negligible number of patients remains undertreated. We found enrollment in Asia to be a strong predictor of prescription pattern. The low prescription rate of OAC treatment in Asia may be due to several reasons. For example, it has been reported that the risk of bleeding during anticoagulation treatment with VKA is increased among Asian patients [22, 23]. The bleeding risk was not increased with NOAC treatment [22]. In addition, in GLORIA-AF most patients from Asia were enrolled in China, where VKA treatment is often not prescribed because international normalized ratio control of VKA therapy is not easily accessible [24, 25]. Within Asia, China had the highest proportion of AP treatment (see Fig 4; China, 37.4% and Asia overall, 24.3%) and no treatment (China, 21.8% and Asia overall, 14.1%). Reimbursement for NOACs in China is recent, which may explain the low prescription. Although the effect of region declined in phase III compared with phase II, it still played a role in prescription. In phase II, patients recruited in Asia, North America, and Latin America were more likely to be prescribed with no OAC treatment versus OAC treatment compared with Europe. In phase III, only Asia was strongly associated with no OAC treatment (vs. Europe). In addition to reimbursement status, treatment availability might have impacted regional prescribing differences, for example only dabigatran and rivaroxaban were approved in China during study conduct, while apixaban was not. Furthermore, the observed regional differences could be linked to ethnic factors and highlight the need to include different populations early in the drug development process, as stated in the International Council for Harmonisation (ICH) E5 and ICH E17 guidelines. Additionally, a high bleeding risk defined by the HAS-BLED score (≥3 vs. <3) and factors increasing the bleeding risk (e.g., prior bleeding and abnormal kidney function) were associated with no OAC prescription. The stable proportion of patients on AP treatment alone or no OAC treatment remained at more than 11% and 6% of patients in phase III, respectively, despite the increased evidence on the superiority of NOACs and VKA in reducing the stroke risk compared to no OAC with no differences in the bleeding risk with VKA, NOAC, or AP therapy [6], emphasizes that there is still a high unmet medical need for a remarkable number of patients. As treatment initiation varies broadly between region and site, clear and consistent guidelines with AF treatment pathways, along with further education and awareness, would improve patient outcomes and reduce health care costs [26].

In both study phases II and III, one of the predictors of OAC treatment was an increased stroke risk, with patients that had a high CHA_2_DS_2_-VASc score (CHA_2_DS_2_-VASc ≥2 for male patients or ≥3 for female patients) being more likely to be prescribed OACs than patients with a low risk score. The CHA_2_DS_2_-VASc score is recommended by the European Society of Cardiology Guidelines and the AHA/ACC/HRS Guidelines for the Management of Patients with AF as a tool to assess risk of stroke in AF patients [6, 18]. Furthermore, several comorbidities that increase stroke risk, such as prior stroke/transient ischemic attack/systemic embolism, hypertension, vascular disease, and diabetes mellitus, were identified as independent predictors of OAC treatment.

In contrast to the recommendations of the AHA/ACC/HRS guidelines, which state that the choice of ATT should be irrespective of the type of AF [19], paroxysmal and persistent AF increased the probability of no OAC prescription compared with permanent AF. This finding may be explained by the perception that permanent AF is more hazardous, although it was shown that the types have similar stroke risks [27].

Decision-making on antithrombotic therapy needs to weigh the risk of stroke against the increased bleeding risk. In our study, we found a high bleeding risk (HAS-BLED score ≥3 vs. <3) was associated with the prescription of no OAC treatment.

Both physician specialty and study site were associated with the prescription decision. Variations in the adoption of new drugs by different physician specialties have been described previously [28]. Patients treated by a general practitioner/primary care physician/geriatrician were less likely to receive an OAC treatment. In line with findings from previous studies, we found that neurologists especially were more likely to prescribe NOACs, whereas internists more often prescribed VKAs compared with cardiologists [29]. Interestingly, we found a change in the prescription pattern of VKA treatment in specialist offices between both study phases. Whilst treatment in specialist offices was associated with a lower prescription of VKA in phase II, the effect was inverted in phase III. Besides these factors, an impaired kidney function was associated with a preference to prescribe VKA over NOACs in phase II and phase III. Stroke risk score was consistently associated with preference of NOAC treatment in both phase II (VKA vs. NOAC: moderate vs. low risk score 0.85, 95% CI 0.74–0.99; high vs. low risk score 0.85, 95% CI 0.74–0.98) and phase III (VKA vs. NOAC: moderate vs. low risk score 0.85, 95% CI 0.70–1.03; high vs. low risk score 0.87, 95% CI 0.72–1.05).

### Limitations and strengths

The comparison between phase II and phase III might have been affected by the decision to begin phase III when patient comparability was reached, rather than by year. As a result, phase III enrollment in each region started at different time points after phase II. Representation of some regions such as Latin America, which enrolled comparatively few patients (7.7% of study participants), is limited. Because many countries enrolled low numbers of patients, countries were grouped into regions for the analyses. Moreover, cluster analyses illustrated that countries from the same region may not have the same treatment patterns. However, cluster analyses do not account for differences in patient characteristics that may partially explain differences in treatment patterns. In addition, due to the aim of GLORIA-AF to assess NOACs and compare dabigatran with VKA, eligibility criteria mirrored dabigatran’s indication for use, by limiting the enrollment to patients with a CHA_2_DS_2_-VASc score of ≥1. Therefore, no data on patients at a very low risk (CHA_2_DS_2_-VASc score of 0) were available. Despite these limitations, GLORIA-AF is one of the largest long-term global registry programs focused on anticoagulation therapy for stroke prevention in patients with nonvalvular AF. The participating sites were included in GLORIA-AF based on the goal to represent the proportion of patients treated within the different healthcare settings of the respective country. Furthermore, with a follow-up time of 2 years in phase II and 3 years in phase III, GLORIA-AF overcomes the limitations of several previous registries that were limited by a short follow-up period. High data quality is ensured by periodic site monitoring, independent audits, bimonthly telephone calls, multiple automatic data checks, bimonthly manual data reviews, and quarterly medical quality reviews of aggregate data. The registry provides valuable data on the ATT of newly diagnosed nonvalvular AF patients in clinical practice around the world. By design, selection bias on a site level and patient level was reduced by the consecutive enrollment of eligible patients at various sites around the world [12].

## Conclusion

Data from phase III of GLORIA-AF showed that the majority of patients with newly diagnosed nonvalvular AF at risk of stroke were treated with OACs, with an increase in use of NOACs and a decrease in VKAs over the past few years. However, in some settings, patients remained undertreated, especially in some countries in Asia. One important predictor of OAC treatment was a high stroke risk score (CHA_2_DS_2_-VASc score ≥2 for male or ≥3 for female), whereas a high bleeding risk decreased the probability of receiving OAC treatment. While the prescription of NOACs was generally preferred over VKAs, impaired kidney function was associated with a preference for VKAs. In addition to clinical factors, other factors such as the care setting, region, and physician specialty were also associated with treatment decisions.

## Supporting information

S1 File Baseline predictor phase III. (DOCX)
