# Supplementary material for: Evolution of antithrombotic therapy for patients with atrial fibrillation: The prospective global GLORIA-AF registry program
Source: PLoS One. 2022 Oct 6;17(10):e0274237. doi: 10.1371/journal.pone.0274237 (PMC9536607; doi:10.1371/journal.pone.0274237)
Supplement: S1 File — (DOCX) [file pone.0274237.s001.docx]

Evolution of antithrombotic therapy for patients with atrial fibrillation: The prospective global GLORIA-AF registry program

Lea Beier, Shihai Lu, Lionel Riou França, Sabrina Marler, Gregory Y H Lip, Menno V Huisman, Christine Teutsch, Jonathan L Halperin, Kristina Zint, Hans-Christoph Diener, Laurie Baker, Chang Sheng Ma, Miney Paquette, Dorothee B Bartels, Sergio J Dubner, Philippe Lyrer, Jochen Senges, Kenneth J Rothman

# Supporting information

**S1 Table.** **Additional baseline characteristics by ATT for phase III patients enrolled between 2014 and 2016.**

**S2 Table. Baseline characteristics by study phase: All eligible patients.**

**S3 Table.** **Factors associated with prescription of no OAC versus OAC using multiple imputation – Model 1.**

**S4 Table.** **Factors associated with prescription of no OAC versus OAC using multiple imputation – Model 2.**

**S5 Table.** **Factors associated with prescription of VKA versus NOAC using multiple imputation – Model 1.**

**S6 Table.** **Factors associated with prescription of VKA versus NOAC using multiple imputation – Model 2.**

**S7 Table.** **Factors associated with prescription of AP versus no OAC using multiple imputation – Model 1.**

**S8 Table.** **Factors associated with prescription of AP versus no OAC using multiple imputation – Model 2.**

**S1 Table. Additional** **baseline characteristics by ATT for phase III patients enrolled between 2014 and 2016.**

| **N (%)** | **All**  **N = 21,241** | **NOAC (± AP)**  **N = 12,637** | **VKA (± AP)**  **N = 4828** | **AP**  **N = 2373** | **No ATT**  **N = 1403** |
| --- | --- | --- | --- | --- | --- |
| Patient characteristics |  |  |  |  |  |
| Age ≥75 years | 8135 (38.3) | 4976 (39.4) | 1949 (40.4) | 771 (32.5) | 439 (31.3) |
| Smoking status |  |  |  |  |  |
| Nonsmoker | 12,154 (57.2) | 7143 (56.5) | 2756 (57.1) | 1401 (59.0) | 854 (60.9) |
| Current smoker | 2027 (9.5) | 1105 (8.7) | 444 (9.2) | 321 (13.5) | 157 (11.2) |
| Past smoker | 6430 (30.3) | 3979 (31.5) | 1488 (30.8) | 618 (26.0) | 345 (24.6) |
| Unknown | 630 (3.0) | 410 (3.2) | 140 (2.9) | 33 (1.4) | 47 (3.3) |
| Creatinine clearance,^*^ mL/min |  |  |  |  |  |
| <15 | 142 (0.7) | 59 (0.5) | 53 (1.1) | 20 (0.8) | 10 (0.7) |
| 15 to <30 | 397 (1.9) | 152 (1.2) | 158 (3.3) | 63 (2.7) | 24 (1.7) |
| 30 to <50 | 2373 (11.2) | 1355 (10.7) | 602 (12.5) | 264 (11.1) | 152 (10.8) |
| 50 to <80 | 6677 (31.4) | 4104 (32.5) | 1473 (30.5) | 691 (29.1) | 409 (29.2) |
| ≥80 | 7454 (35.1) | 4581 (36.3) | 1527 (31.6) | 855 (36.0) | 491 (35.0) |
| Medical treatment reimbursement |  |  |  |  |  |
| Private insurance | 3084 (14.5) | 2063 (16.3) | 489 (10.1) | 362 (15.3) | 170 (12.1) |
| Statutory/federal insurance | 15,723 (74.0) | 9062 (71.7) | 3811 (78.9) | 1782 (75.1) | 1068 (76.1) |
| Self-pay/no coverage | 1015 (4.8) | 624 (4.9) | 221 (4.6) | 112 (4.7) | 58 (4.1) |

AP, antiplatelets; ATT, antithrombotic treatment; NOAC, novel/nonvitamin K oral anticoagulants; VKA, vitamin K antagonist.

^*^Data missing for 4198 (19.8%) overall patients.

**S2 Table. Baseline characteristics by study phase: All eligible patients.**

| **N (%) or mean ± SD** | **Phase II***  **N = 14,704** | **Phase III**  **N = 21,241** | **Standardized Difference** |
| --- | --- | --- | --- |
| Patient characteristics |  |  |  |
| Age, years | 70.6 ± 10.9 | 70.5 ± 10.6 | 0.0052 |
| ≥75 | 5799 (39.4) | 8135 (38.3) | 0.0234 |
| Sex, female | 6678 (45.4) | 9546 (44.9) | 0.0095 |
| BMI, kg/m²^†^ | 28.5 ± 6.1 | 28.6 ± 6.4 | –0.0130 |
| Smoking status |  |  |  |
| Nonsmoker | 8469 (57.6) | 12,154 (57.2) | 0.0076 |
| Current smoker | 1369 (9.3) | 2027 (9.5) | –0.0080 |
| Past smoker | 4332 (29.5) | 6430 (30.3) | –0.0177 |
| Unknown | 534 (3.6) | 630 (3.0) | 0.0373 |
| Medical history |  |  |  |
| Hypertension |  |  |  |
| Uncontrolled | 1692 (11.5) | 2157 (10.2) | 0.0435 |
| Controlled | 9030 (61.4) | 13,358 (62.9) | –0.0304 |
| Congestive heart failure | 3494 (23.8) | 4616 (21.7) | 0.0485 |
| Diabetes mellitus | 3279 (22.3) | 4940 (23.3) | –0.0228 |
| Stroke/transient ischemic attack/ systemic embolism | 2163 (14.7) | 3086 (14.5) | 0.0051 |
| Vascular disease^‡^ | 1969 (13.4) | 2691 (12.7) | 0.0214 |
| Creatinine clearance,^§^ mL/min |  |  |  |
| <15 | 103 (0.7) | 142 (0.7) | 0.0039 |
| 15 to <30 | 294 (2.0) | 397 (1.9) | 0.0095 |
| 30 to <50 | 1755 (11.9) | 2373 (11.2) | 0.0239 |
| 50 to <80 | 4542 (30.9) | 6677 (31.4) | –0.0118 |
| ≥80 | 4900 (33.3) | 7454 (35.1) | –0.0373 |
| Cancer | 1406 (9.6) | 2112 (9.9) | –0.0128 |
| Chronic gastrointestinal diseases | 1975 (13.4) | 2814 (13.2) | 0.0054 |
| Abnormal kidney function^\|\|^ | 224 (1.5) | 389 (1.8) | –0.0240 |
| Hyperlipidemia | 5838 (39.7) | 8296 (39.1) | 0.0132 |
| Coronary artery disease | 2913 (19.8) | 3967 (18.7) | 0.0288 |
| Transient ischemic attack | 641 (4.4) | 948 (4.5) | –0.0051 |
| AP drug use^¶^ | 3857 (26.2) | 5425 (25.5) | 0.0158 |
| Interventions in AF |  |  |  |
| Cardioversion | 2362 (16.1) | 3840 (18.1) | –0.0536 |
| AF ablation | 157 (1.1) | 382 (1.8) | –0.0615 |
| Region |  |  |  |
| Asia | 3070 (20.9) | 4239 (20.0) | 0.0229 |
| Europe | 7332 (49.9) | 10,279 (48.4) | 0.0294 |
| North America | 3391 (23.1) | 5097 (24.0) | –0.0220 |
| Latin America | 911 (6.2) | 1626 (7.7) | –0.0575 |
| CHA_2_DS_2-_VASc score |  |  |  |
| Low: 1 for women | 324 (2.2) | 488 (2.3) | –0.0063 |
| Moderate: 1 for men or 2 for women | 2759 (18.8) | 3967 (18.7) | 0.0022 |
| High: ≥2 for men or ≥3 for women | 11,621 (79.0) | 16,786 (79.0) | 0.0002 |
| HAS-BLED score ^#^ |  |  |  |
| Low: <3 | 11,604 (78.9) | 17,242 (81.2) | –0.0565 |
| High: ≥3 | 1341 (9.1) | 1970 (9.3) | –0.0053 |
| Type of AF |  |  |  |
| Paroxysmal | 7867 (53.5) | 11,972 (56.4) | –0.0575 |
| Persistent | 5264 (35.8) | 7249 (34.1) | 0.0351 |
| Permanent | 1573 (10.7) | 2020 (9.5) | 0.0394 |
| Categorization of AF |  |  |  |
| Symptomatic | 4155 (28.3) | 6588 (31.0) | –0.0604 |
| Minimally symptomatic | 5824 (39.6) | 7216 (34.0) | 0.1171 |
| Asymptomatic | 4725 (32.1) | 7437 (35.0) | –0.0610 |
| Physician specialty^**^ |  |  |  |
| GP/PCP/geriatrician | 501 (3.4) | 1055 (5.0) | –0.0779 |
| Cardiologist | 12,905 (87.8) | 18,056 (85.0) | 0.0805 |
| Neurologist | 402 (2.7) | 524 (2.5) | 0.0168 |
| Internist | 425 (2.9) | 820 (3.9) | –0.0537 |
| Other | 456 (3.1) | 779 (3.7) | –0.0313 |
| Medical treatment reimbursement |  |  |  |
| Private insurance | 2146 (14.6) | 3084 (14.5) | 0.0021 |
| Statutory/federal insurance | 10,703 (72.8) | 15,723 (74.0) | –0.0279 |
| Self-pay/no coverage | 973 (6.6) | 1015 (4.8) | 0.0794 |
| Type of site |  |  |  |
| GP/primary care | 970 (6.6) | 1318 (6.2) | 0.0160 |
| Specialist office | 4425 (30.1) | 6216 (29.3) | 0.0182 |
| Community hospital | 3972 (27.0) | 6252 (29.4) | –0.0538 |
| University hospital | 4900 (33.3) | 6756 (31.8) | 0.0324 |
| Outpatient healthcare center | 240 (1.6) | 335 (1.6) | 0.0044 |
| Anticoagulation clinics | 71 (0.5) | 118 (0.6) | –0.0101 |
| Other | 126 (0.9) | 246 (1.2) | –0.0302 |
| ATT prescription |  |  |  |
| NOACs | 6994 (47.6) | 12,637 (59.5) | –0.2409 |
| VKA | 4773 (32.5) | 4828 (22.7) | 0.2190 |
| APs alone | 1776 (12.1) | 2373 (11.2) | 0.0283 |
| No ATT | 1161 (7.9) | 1403 (6.6) | 0.0498 |

AF, atrial fibrillation; AP, antiplatelets; ATT, antithrombotic treatment; BMI, body mass index; CHA_2_DS_2_-VASc, congestive heart failure, hypertension, age ≥75 years, diabetes, stroke/transient ischemic attack/systemic embolism, vascular disease, age from 65–74 years, sex category (female); GP, general practitioner; HAS-BLED, hypertension, abnormal renal/liver function, stroke, bleeding history or predisposition, labile international normalized ratio, elderly (>65 years), drugs or alcohol concomitantly; NOAC, novel/nonvitamin K oral anticoagulants; PCP, primary care physician; SD, standard deviation; VKA, vitamin K antagonist.

*Excluding patients from Africa/the Middle East.

^†^Data missing for 192 (1.3%) phase II and 245 (1.2%) phase III patients.

^‡^Defined as prior myocardial infarction, peripheral artery disease, complex aortic plaque.

^§^Data missing for 3110 (21.2%) phase II and 4198 (19.8%) phase III patients.

^||^Defined as presence of chronic dialysis or renal transplantation or serum creatinine ≥200 μmol/L.

^¶^Defined as AP use on baseline visit.

^#^Data missing for 1759 (12.0%) phase II and 2029 (9.6%) phase III patients.

^**^Three patients in phase III were enrolled by an angiologist.

**S3 Table. Factors associated with prescription of no OAC versus OAC using multiple imputation – Model 1.**

| **Factor** | **Multivariate analysis relative risk estimates from the log-binomial regression analysis** | | | | | |
| --- | --- | --- | --- | --- | --- | --- |
|  | **Phase II*** | | | **Phase III** | | |
|  | **Total N** | **No OAC vs. OAC**^†^**, n (%)** | **Relative proportion  (95% CI)**^‡^ | **Total N** | **No OAC vs. OAC**^†^**, n (%)** | **Relative proportion  (95% CI)**^‡^ |
| **BMI, kg/m^2^**  <18.5   18.5 to <25   25 to <30  30 to <35   ≥35 | 220  4027 5691  2923  1844 | 60 (27.3) vs. 159 (72.3)  1094 (27.2) vs. 2933 (72.8) 1102 (19.4) vs. 4589 (80.6)  438 (15.0) vs. 2485 (85.0)  243 (13.2) vs. 1601 (86.8) | 1.019 (0.870, 1.194) 1.0 (ref) 0.963 (0.924, 1.003) 0.884 (0.806, 0.969) 0.717 (0.633, 0.813) | 284  5950  8052  4184 2771 | 72 (25.4) vs. 212 (74.6)  1428 (24.0) vs. 4521 (76.0)  1385 (17.2) vs. 6667 (82.8)  570 (13.6) vs. 3614 (86.4)  320 (11.5) vs. 2451 (88.5) | 1.070 (0.994, 1.152) 1.0 (ref) 0.998 (0.962, 1.037) 0.952 (0.881, 1.029) 0.856 (0.764, 0.960) |
| **Region**  Asia   Europe   North America   Latin America | 3070 7332  3391  911 | 1374 (44.8) vs. 1696 (55.2)  708 (9.7) vs. 6624 (90.3)  724 (21.4) vs. 2667 (78.6) 131 (14.4) vs. 780 (85.6) | 2.568 (2.350, 2.805) 1.0 (ref) 1.752 (1.585, 1.937) 1.318 (1.115, 1.558) | 4239 10,279 5097 1626 | 1630 (38.5) vs. 2609 (61.5) 1097 (10.7) vs. 9182 (89.3) 836 (16.4) vs. 4261 (83.6) 213 (13.1) vs. 1413 (86.9) | 1.942 (1.802, 2.094) 1.0 (ref) 1.007 (0.922, 1.100) 1.137 (0.996, 1.298) |
| **CHA_2_DS_2_-VASc score**  Low: 1 for women   Moderate: 1 for men or 2 for women   High: ≥2 for men or ≥3 for women | 324  2759 11,621 | 176 (54.3) vs. 148 (45.7)  801 (29.0) vs. 1958 (71.0)  1960 (16.9) vs. 9661 (83.1) | 1.0 (ref) 0.943 (0.911, 0.975) 0.749 (0.701, 0.801) | 488 3967 16,786 | 247 (50.6) vs. 241 (49.4)  979 (24.7) vs. 2988 (75.3) 2550 (15.2) vs. 14,236 (84.8) | 1.0 (ref) 0.979 (0.936, 1.025) 0.779 (0.733, 0.828) |
| **HAS-BLED (imputed) risk score**  Low: <3   High: ≥3 | 13,189 1515 | 2388 (18.1) vs. 10,801 (81.9)  549 (36.2) vs. 966 (63.8) | 1.0 (ref) 1.150 (1.092, 1.211) | 19,032 2209 | 3010 (15.8) vs. 16,022 (84.2) 766 (34.7) vs. 1443 (65.3) | 1.0 (ref) 1.163 (1.109, 1.220) |
| **Type of AF**  Paroxysmal   Persistent   Permanent | 7867  5264 1573 | 1944 (24.7) vs. 5923 (75.3)  859 (16.3) vs. 4405 (83.7)  134 (8.5) vs. 1439 (91.5) | 1.695 (1.444, 1.990) 1.493 (1.268, 1.758) 1.0 (ref) | 11,972 7249 2020 | 2653 (22.2) vs. 9319 (77.8) 948 (13.1) vs. 6301 (86.9) 175 (8.7) vs. 1845 (91.3) | 1.519 (1.319, 1.749) 1.271 (1.100, 1.468) 1.0 (ref) |
| **Categorization of AF**  Symptomatic   Minimally symptomatic   Asymptomatic | 4155 5824 4725 | 748 (18.0) vs. 3407 (82.0)  1264 (21.7) vs. 4560 (78.3)  925 (19.6) vs. 3800 (80.4) | 0.987 (0.932, 1.044) 1.018 (0.980, 1.058) 1.0 (ref) | 6588 7216 7437 | 1183 (18.0) vs. 5405 (82.0) 1393 (19.3) vs. 5823 (80.7) 1200 (16.1) vs. 6237 (83.9) | 1.020 (0.984, 1.057) 1.032 (0.992, 1.074) 1.0 (ref) |
| **AF cardioversion**  Yes   No | 2388 12,316 | 412 (17.3) vs. 1976 (82.7)  2525 (20.5) vs. 9791 (79.5) | 0.946 (0.911, 0.981) 1.0 (ref) | 3872 17,369 | 662 (17.1) vs. 3210 (82.9) 3114 (17.9) vs. 14,255 (82.1) | 1.012 (0.990, 1.035) 1.0 (ref) |
| **Creatinine clearance, mL/min**  <30   30 to <50   50 to <80   ≥80 | 470  2307  5731 6195 | 117 (24.9) vs. 353 (75.1)  472 (20.5) vs. 1835 (79.5)  1112 (19.4) vs. 4620 (80.6)  1236 (20.0) vs. 4960 (80.1) | 1.117 (1.013, 1.230) 0.980 (0.912, 1.054) 0.941 (0.900, 0.984) 1.0 (ref) | 642 3086 8258 9256 | 136 (21.2) vs. 505 (78.7)  529 (17.1) vs. 2556 (82.8) 1384 (16.8) vs. 6874 (83.2) 1726 (18.6) vs. 7530 (81.4) | 0.997 (0.917, 1.083) 0.993 (0.915, 1.078) 0.961 (0.912, 1.014) 1.0 (ref) |
| **Cancer**  Yes   No | 1417 13,287 | 237 (16.7) vs. 1181 (83.3)  2700 (20.3) vs. 10,586 (79.7) | 0.989 (0.887, 1.103) 1.0 (ref) | 2142 19,100 | 321 (15.0) vs. 1821 (85.0) 3455 (18.1) vs. 15,644 (81.9) | 0.974 (0.889, 1.067) 1.0 (ref) |
| **Chronic gastrointestinal disease**  Yes  No | 1996 12,708 | 388 (19.4) vs. 1608 (80.6)  2549 (20.1) vs. 10,159 (79.9) | 0.939 (0.891, 0.990) 1.0 (ref) | 2857 18,384 | 513 (18.0) vs. 2343 (82.0) 3263 (17.7) vs. 15,122 (82.3) | 1.020 (0.983, 1.057) 1.0 (ref) |
| **Hyperlipidemia**  Yes  No | 5966 8738 | 942 (15.8) vs. 5024 (84.2)  1995 (22.8) vs. 6743 (77.2) | 0.774 (0.722, 0.829) 1.0 (ref) | 8454 12,787 | 1202 (14.2) vs. 7251 (85.8) 2574 (20.1) vs. 10,214 (79.9) | 0.799 (0.751, 0.850) 1.0 (ref) |
| **Coronary artery disease**  Yes  No | 2961 11,743 | 732 (24.7) vs. 2229 (75.3)  2205 (18.8) vs. 9538 (81.2) | 1.035 (0.980, 1.093) 1.0 (ref) | 4025 17,216 | 927 (23.0) vs. 3099 (77.0) 2849 (16.5) vs. 14,366 (83.4) | 0.944 (0.906, 0.983) 1.0 (ref) |
| **Smoking status**  Nonsmoker   Current smoker  Past smoker | 8802  1416 4486 | 1788 (20.3) vs. 7014 (79.7)  405 (28.6) vs. 1011 (71.4)  744 (16.6) vs. 3742 (83.4) | 1.0 (ref) 0.993 (0.946, 1.042) 0.901 (0.843, 0.963) | 12,538 2089 6614 | 2301 (18.4) vs. 10,237 (81.6) 491 (23.5) vs. 1598 (76.5) 984 (14.9) vs. 5630 (85.1) | 1.0 (ref) 0.992 (0.937, 1.049) 0.924 (0.886, 0.964) |
| **AP drug use**^§^  Yes   No | 3857 10,847 | 1759 (45.6) vs. 2098 (54.4)  1178 (10.9) vs. 9669 (89.1) | 2.968 (2.771, 3.179) 1.0 (ref) | 5425 15,816 | 2372 (43.7) vs. 3053 (56.3) 1404 (8.9) vs. 14,412 (91.1) | 4.157 (3.907, 4.424) 1.0 (ref) |
| **Physician specialty**  GP/PCP/geriatrician   Cardiologist   Neurologist   Internist   Other | 501  12,918  402  425  458 | 114 (22.8) vs. 387 (77.2)  2638 (20.4) vs. 10,280 (79.6)  51 (12.7) vs. 351 (87.3)  70 (16.5) vs. 355 (83.5)  64 (14.0) vs. 394 (86.0) | 1.199 (1.120, 1.284) 1.0 (ref) 0.974 (0.759, 1.250) 1.119 (0.914, 1.369) 1.129 (0.929, 1.371) | 1058 18,057 524 820 782 | 256 (24.2) vs. 802 (75.8) 3277 (18.1) vs. 14,780 (81.9) 71 (13.5) vs. 453 (86.5)  82 (10.0) vs. 738 (90.0) 90 (11.5) vs. 692 (88.5) | 1.069 (1.015, 1.126) 1.0 (ref) 1.027 (0.844, 1.251) 0.894 (0.733, 1.090) 0.964 (0.802, 1.158) |
| **Medical treatment reimbursement**   Self-pay/no coverage  Not self-pay | 1041  13,663 | 274 (26.3) vs. 767 (73.7)  2663 (19.5) vs. 11,000 (80.5) | 1.072 (1.031, 1.115) 1.0 (ref) | 1077 20,164 | 180 (16.7) vs. 897 (83.3) 3596 (17.8) vs. 16,568 (82.2) | 0.983 (0.869, 1.112) 1.0 (ref) |
| **Type of site**  Specialist office  Community hospital   University hospital   Other^\|\|^ | 4425  3972  4900 1407 | 911 (20.6) vs. 3514 (79.4)  531 (13.4) vs. 3441 (86.6)  1198 (24.4) vs. 3702 (75.6)  297 (21.1) vs. 1110 (78.9) | 1.105 (1.017, 1.202) 1.0 (ref) 1.036 (0.955, 1.125) 1.116 (1.016, 1.225) | 6216 6252 6756 2017 | 1019 (16.4) vs. 5197 (83.6) 842 (13.5) vs. 5410 (86.5) 1407 (20.8) vs. 5349 (79.2) 508 (25.2) vs. 1509 (74.8) | 1.094 (1.020, 1.173) 1.0 (ref) 1.108 (1.043, 1.176) 1.122 (1.058, 1.189) |

AF, atrial fibrillation; AP, antiplatelets; ASA, acetylsalicylic acid; BMI, body mass index; CHA_2_DS_2_-VASc, congestive heart failure, hypertension, age ≥75 years, diabetes, stroke/transient ischemic attack/systemic embolism, vascular disease, age from 65–74 years, sex category (female); CI, confidence interval; F, female; GP, general practitioner; HAS-BLED, hypertension, abnormal renal/liver function, stroke, bleeding history or predisposition, labile international normalized ratio, elderly (>65 years), drugs or alcohol concomitantly; M, male; OAC, oral anticoagulant; PCP, primary care physician; ref, reference; VKA, vitamin K antagonist.

^*^Excluding patients from Africa/the Middle East.

^†^The data of patients in combinations of oral anticoagulants treatment group are not considered. OAC includes dabigatran; VKA, rivaroxaban and apixaban. No OAC use includes ASA, APs other than ASA, and none.

^‡^CIs were calculated based on the likelihood method.

^§^AP use was defined as use at the baseline visit.

^||^GP/primary care, outpatient healthcare centre, anticoagulation clinics, and other.

**S4 Table.** **Factors associated with prescription of no OAC versus OAC using multiple imputation – Model 2**.

| **Factor** | **Multivariate analysis relative risk estimates from the log-binomial regression analysis** | | | | | |
| --- | --- | --- | --- | --- | --- | --- |
|  | **Phase II*** | | | **Phase III** | | |
|  | **Total N** | **No OAC vs. OAC**^†^**, n (%)** | **Relative proportion (95% CI)**^‡^ | **Total N** | **No OAC vs. OAC**^†^**, n (%)** | **Relative proportion  (95% CI)**^‡^ |
| **BMI, kg/m^2^**  <18.5   18.5 to <25   25 to <30  30 to <35   ≥35 | 220 4027 5691 2923 1844 | 60 (27.3) vs. 159 (72.3)  1094 (27.2) vs. 2933 (72.8)  1102 (19.4) vs. 4589 (80.6)  438 (15.0) vs. 2485 (85.0)  243 (13.2) vs. 1601 (86.8) | 0.954 (0.812, 1.120) 1.0 (ref) 0.967 (0.928, 1.008) 0.889 (0.805, 0.983) 0.729 (0.641, 0.829) | 284 5950 8052 4184 2771 | 72 (25.4) vs. 212 (74.6) 1428 (24.0) vs. 4521 (76.0) 1385 (17.2) vs. 6667 (82.8) 570 (13.6) vs. 3614 (86.4) 320 (11.5) vs. 2451 (88.5) | 1.134 (1.032, 1.247) 1.0 (ref) 0.999 (0.960, 1.040) 0.969 (0.896, 1.047) 0.869 (0.774, 0.974) |
| **Region**  Asia   Europe   North America   Latin America | 3070 7332 3391 911 | 1374 (44.8) vs. 1696 (55.2) 708 (9.7) vs. 6624 (90.3) 724 (21.4) vs. 2667 (78.6) 131 (14.4) vs. 780 (85.6) | 2.584 (2.365, 2.822) 1.0 (ref) 1.698 (1.534, 1.879) 1.285 (1.087, 1.518) | 4239 10,279 5097 1626 | 1630 (38.5) vs. 2609 (61.5) 1097 (10.7) vs. 9182 (89.3) 836 (16.4) vs. 4261 (83.6) 213 (13.1) vs. 1413 (86.9) | 1.954 (1.811, 2.109) 1.0 (ref) 0.983 (0.900, 1.075) 1.132 (0.993, 1.291) |
| **Congestive heart failure/LV dysfunction**  Yes  No | 3531 11,173 | 665 (18.8) vs. 2866 (81.2)  2272 (20.3) vs. 8901 (79.7) | 0.972 (0.921, 1.026) 1.0 (ref) | 4653 16,588 | 762 (16.4) vs. 3892 (83.6) 3014 (18.2) vs. 13,574 (81.8) | 0.958 (0.907, 1.012) 1.0 (ref) |
| **History of hypertension**  Yes  No | 10,950 3754 | 2006 (18.3) vs. 8945 (81.7)  931 (24.8) vs. 2822 (75.2) | 0.925 (0.884, 0.968) 1.0 (ref) | 15,862 5379 | 2554 (16.1) vs. 13,308 (83.9) 1222 (22.7) vs. 4157 (77.3) | 0.910 (0.873, 0.948) 1.0 (ref) |
| **Abnormal kidney function**  Yes  No | 228 14,477 | 73 (32.0) vs. 154 (67.5)  2864 (19.8) vs. 11,613 (80.2) | 1.161 (0.962, 1.401) 1.0 (ref) | 395 20,846 | 112 (28.4) vs. 283 (71.6) 3664 (17.6) vs. 17,182 (82.4) | 1.242 (1.124, 1.373) 1.0 (ref) |
| **Diabetes mellitus**  Yes  No | 3279 11,425 | 566 (17.3) vs. 2713 (82.7)  2371 (20.8) vs. 9054 (79.2) | 0.910 (0.850, 0.974) 1.0 (ref) | 4940 16,301 | 780 (15.8) vs. 4160 (84.2) 2996 (18.4) vs. 13,305 (81.6) | 0.932 (0.878, 0.989) 1.0 (ref) |
| **Stroke/TIA/systemic embolism**  Yes  No | 646 14,058 | 89 (13.8) vs. 557 (86.2)  2849 (20.3) vs. 11,210 (79.7) | 0.841 (0.700, 1.009) 1.0 (ref) | 956 20,285 | 115 (12.0) vs. 841 (88.0) 3661 (18.0) vs. 16,624 (82.0) | 0.820 (0.696, 0.965) 1.0 (ref) |
| **Vascular disease**^§^  Yes  No | 1969 12,735 | 423 (21.5) vs. 1546 (78.5) 2514 (19.7) vs. 10,221 (80.3) | 0.940 (0.871, 1.016) 1.0 (ref) | 2691 18,550 | 549 (20.4) vs. 2142 (79.6) 3227 (17.4) vs. 15,323 (82.6) | 0.914 (0.847, 0.985) 1.0 (ref) |
| **Age**  <65   65 to <75  ≥75 | 3915 4990 5799 | 1051 (26.8) vs. 2864 (73.2) 920 (18.4) vs. 4070 (81.6) 966 (16.7) vs. 4833 (83.3) | 1.061 (1.023, 1.101) 1.008 (0.951, 1.069) 1.0 (ref) | 5414 7692 8135 | 1354 (25.0) vs. 4060 (75.0) 1212 (15.8) vs. 6480 (84.2) 1210 (14.9) vs. 6925 (85.1) | 1.101 (1.031, 1.175) 0.998 (0.941, 1.059) 1.0 (ref) |
| **Sex**  Male  Female | 8026 6678 | 1579 (19.7) vs. 6447 (80.3) 1358 (20.3) vs. 5320 (79.7) | 1.0 (ref) 1.026 (0.974, 1.082) | 11,695 9546 | 2081 (17.8) vs. 9614 (82.2) 1695 (17.8) vs. 7851 (82.2) | 1.0 (ref) 1.022 (0.979, 1.066) |
| **Hepatic disease**  Yes  No | 248 14,456 | 64 (25.8) vs. 184 (74.2)  2873 (19.9) vs. 11,583 (80.1) | 1.095 (0.935, 1.284) 1.0 (ref) | 323 20,918 | 77 (23.8) vs. 246 (76.2) 3699 (17.7) vs. 17,219 (82.3) | 0.932 (0.826, 1.051) 1.0 (ref) |
| **Prior bleeding**  Yes  No | 839 13,593 | 188 (22.4) vs. 651 (77.6) 2718 (20.0) vs. 10,875 (80.0) | 1.199 (1.083, 1.327) 1.0 (ref) | 1124 20,117 | 262 (23.3) vs. 862 (76.7) 3514 (17.5) vs. 16,603 (82.5) | 1.121 (1.060, 1.187) 1.0 (ref) |
| **Alcohol abuse**  Yes  No | 1114 13,591 | 200 (18.0) vs. 913 (82.0) 2737 (20.1) vs. 10,854 (79.9) | 0.952 (0.865, 1.047) 1.0 (ref) | 1573 19,668 | 241 (15.3) vs. 1331 (84.6) 3535 (18.0) vs. 16,134 (82.0) | 0.998 (0.926, 1.075) 1.0 (ref) |
| **Type of AF**  Paroxysmal   Persistent   Permanent | 7867 5264 1573 | 1944 (24.7) vs. 5923 (75.3)  859 (16.3) vs. 4405 (83.7)  134 (8.5) vs. 1439 (91.5) | 1.728 (1.473, 2.027) 1.496 (1.271, 1.761) 1.0 (ref) | 11,972 7249 2020 | 2653 (22.2) vs. 9319 (77.8) 948 (13.1) vs. 6301 (86.9) 175 (8.7) vs. 1845 (91.3) | 1.538 (1.336, 1.772) 1.285 (1.110, 1.486) 1.0 (ref) |
| **Categorization of AF**  Symptomatic   Minimally symptomatic   Asymptomatic | 4155 5824 4725 | 748 (18.0) vs. 3407 (82.0) 1264 (21.7) vs. 4560 (78.3) 925 (19.6) vs. 3800 (80.4) | 0.977 (0.923, 1.036) 1.010 (0.969, 1.053) 1.0 (ref) | 6588 7216 7437 | 1183 (18.0) vs. 5405 (82.0) 1393 (19.3) vs. 5823 (80.7) 1200 (16.1) vs. 6237 (83.9) | 1.006 (0.958, 1.056) 1.013 (0.965, 1.064) 1.0 (ref) |
| **AF cardioversion**  Yes   No | 2388 12,316 | 412 (17.3) vs. 1976 (82.7)  2525 (20.5) vs. 9791 (79.5) | 0.958 (0.905, 1.014) 1.0 (ref) | 3872 17,369 | 662 (17.1) vs. 3210 (82.9) 3114 (17.9) vs. 14,255 (82.1) | 1.008 (0.987, 1.029) 1.0 (ref) |
| **Creatinine clearance, mL/min**  <30   30 to <50   50 to <80   ≥80 | 470 2307 5731 6195 | 117 (24.9) vs. 353 (75.1)  472 (20.5) vs. 1835 (79.5)  1112 (19.4) vs. 4620 (80.6)  1236 (20.0) vs. 4960 (80.1) | 0.997 (0.829, 1.199) 0.969 (0.899, 1.043) 0.948 (0.904, 0.994) 1.0 (ref) | 642 3086 8258 9256 | 136 (21.2) vs. 505 (78.7) 529 (17.1) vs. 2556 (82.8) 1384 (16.8) vs. 6874 (83.2) 1726 (18.6) vs. 7530 (81.4) | 1.008 (0.891, 1.141) 0.988 (0.901, 1.082) 0.984 (0.930, 1.040) 1.0 (ref) |
| **Cancer**  Yes   No | 1417 13,287 | 237 (16.7) vs. 1181 (83.3)  2700 (20.3) vs. 10,586 (79.7) | 0.994 (0.893, 1.107) 1.0 (ref) | 2142 19,100 | 321 (15.0) vs. 1821 (85.0) 3455 (18.1) vs. 15,644 (81.9) | 0.974 (0.888, 1.068) 1.0 (ref) |
| **Hyperlipidemia**  Yes  No | 5966 8738 | 942 (15.8) vs. 5024 (84.2)  1995 (22.8) vs. 6743 (77.2) | 0.770 (0.719, 0.824) 1.0 (ref) | 8454 12,787 | 1202 (14.2) vs. 7251 (85.8) 2574 (20.1) vs. 10,214 (79.9) | 0.800 (0.751, 0.851) 1.0 (ref) |
| **Coronary artery disease**  Yes  No | 2961 11,743 | 732 (24.7) vs. 2229 (75.3) 2205 (18.8) vs. 9538 (81.2) | 1.019 (0.974, 1.067) 1.0 (ref) | 4025 17,216 | 927 (23.0) vs. 3099 (77.0) 2849 (16.5) vs. 14,366 (83.4) | 0.965 (0.914, 1.018) 1.0 (ref) |
| **Chronic gastrointestinal disease**  Yes  No | 1996 12708 | 388 (19.4) vs. 1608 (80.6)  2549 (20.1) vs. 10159 (79.9) | 0.927 (0.853, 1.008) 1.0 (ref) | 2857 18,384 | 513 (18.0) vs. 2343 (82.0) 3263 (17.7) vs. 15,122 (82.3) | 1.015 (0.964, 1.068) 1.0 (ref) |
| **Smoking Status**  Nonsmoker   Current smoker  Past smoker | 8802 1416 4486 | 1788 (20.3) vs. 7014 (79.7)  405 (28.6) vs. 1011 (71.4)  744 (16.6) vs. 3742 (83.4) | 1.0 (ref) 1.053 (0.997, 1.113) 0.912 (0.848, 0.982) | 12,538 2089 6614 | 2301 (18.4) vs. 10,237 (81.6) 491 (23.5) vs. 1598 (76.5) 984 (14.9) vs. 5630 (85.1) | 1.0 (ref) 1.029 (0.981, 1.080) 0.972 (0.912, 1.036) |
| **AP drug use**^\|\|^  Yes   No | 3857 10,847 | 1759 (45.6) vs. 2098 (54.4)  1178 (10.9) vs. 9669 (89.1) | 3.118 (2.916, 3.335) 1.0 (ref) | 5425 15,816 | 2372 (43.7) vs. 3053 (56.3) 1404 (8.9) vs. 14,412 (91.1) | 4.332 (4.072, 4.609) 1.0 (ref) |
| **Physician specialty**  GP/PCP/geriatrician   Cardiologist   Neurologist   Internist   Other | 501 12,918 402 425 458 | 114 (22.8) vs. 387 (77.2)  2638 (20.4) vs. 10,280 (79.6)  51 (12.7) vs. 351 (87.3)  70 (16.5) vs. 355 (83.5)  64 (14.0) vs. 394 (86.0) | 1.319 (1.200, 1.450) 1.0 (ref) 0.978 (0.762, 1.255) 1.131 (0.921, 1.388) 1.122 (0.922, 1.366) | 1058 18,057 524 820 782 | 256 (24.2) vs. 802 (75.8) 3277 (18.1) vs. 14,780 (81.9) 71 (13.5) vs. 453 (86.5) 82 (10.0) vs. 738 (90.0)  90 (11.5) vs. 692 (88.5) | 1.101 (1.032, 1.175) 1.0 (ref) 1.020 (0.851, 1.224) 0.903 (0.741, 1.101) 0.982 (0.815, 1.184) |
| **Medical treatment reimbursement**  Self-pay/no coverage  Not self-pay | 1041 13,663 | 274 (26.3) vs. 767 (73.7) 2663 (19.5) vs. 11,000 (80.5) | 1.072 (1.024, 1.121) 1.0 (ref) | 1077 20,164 | 180 (16.7) vs. 897 (83.3) 3596 (17.8) vs. 16,568 (82.2) | 0.954 (0.857, 1.062) 1.0 (ref) |
| **Type of site**  Specialist office  Community hospital   University hospital   Other^¶^ | 4425 3972 4900 1407 | 911 (20.6) vs. 3514 (79.4) 531 (13.4) vs. 3441 (86.6) 1198 (24.4) vs. 3702 (75.6) 297 (21.1) vs. 1110 (78.9) | 1.145 (1.062, 1.236) 1.0 (ref) 1.081 (1.000, 1.168) 1.084 (0.993, 1.183) | 6216 6252 6756 2017 | 1019 (16.4) vs. 5197 (83.6) 842 (13.5) vs. 5410 (86.5) 1407 (20.8) vs. 5349 (79.2) 508 (25.2) vs. 1509 (74.8) | 1.115 (1.041, 1.194) 1.0 (ref) 1.119 (1.062, 1.179) 1.127 (1.070, 1.188) |

AF, atrial fibrillation; AP, antiplatelets; ASA, acetylsalicylic acid; BMI, body mass index; CI, confidence interval; GP, general practitioner; LV, left ventricle; OAC, oral anticoagulation; PCP, primary care physician; ref, reference; TIA, transient ischemic attack; VKA, vitamin K antagonist.

^*^Excluding patients from Africa/the Middle East.

^†^The data of patients in Combinations of oral anticoagulants treatment group are not considered. OAC includes dabigatran, VKA, rivaroxaban, and apixaban. No OAC use includes ASA, APs other than ASA, and none.

^‡^CIs were calculated based on the likelihood method.

^§^Prior myocardial infarction, peripheral artery disease, complex aortic plaque.

^||^AP use was defined as use at the baseline visit.

^¶^GP/primary care, outpatient healthcare centre, anticoagulation clinics, and other.

**S5 Table.** **Factors associated with prescription of VKA versus NOAC using multiple imputation – Model 1**.

| **Factor** | **Multivariate analysis relative risk estimates from the log-binomial regression analysis** | | | | | |
| --- | --- | --- | --- | --- | --- | --- |
|  | **Phase II*** | | | **Phase III** | | |
|  | **Total N** | **VKA vs. NOAC**^†^**, n (%)** | **Relative proportion  (95% CI)**^‡^ | **Total N** | **VKA vs. NOAC** ^†^**, n (%)** | **Relative proportion  (95% CI)**^‡^ |
| **BMI, kg/m^2^**  <18.5   18.5 to <25   25 to <30  30 to <35   ≥35 | 159  2933  4589  2485  1601 | 65 (40.9) vs. 95 (59.7)  1248 (42.6) vs. 1685 (57.4)  1869 (40.7) vs. 2720 (59.3)  971 (39.1) vs. 1514 (60.9)  620 (38.7) vs. 981 (61.3) | 0.951 (0.786, 1.150) 1.0 (ref) 1.045 (0.994, 1.098) 1.052 (0.989, 1.120) 1.095 (1.023, 1.171) | 212  4521  6667 3614 2451 | 60 (28.3) vs. 152 (71.7)  1293 (28.6) vs. 3228 (71.4)  1878 (28.2) vs. 4789 (71.8)  967 (26.8) vs. 2647 (73.2)  630 (25.7) vs. 1822 (74.3) | 0.971 (0.783, 1.205) 1.0 (ref) 1.031 (0.972, 1.093) 1.066 (0.990, 1.147) 1.122 (1.028, 1.224) |
| **Region**  Asia   Europe   North America   Latin America | 1696  6624  2667  780 | 846 (49.9) vs. 850 (50.1)  2763 (41.7) vs. 3861 (58.3)  897 (33.6) vs. 1770 (66.4)  267 (34.2) vs. 513 (65.8) | 1.151 (1.093, 1.213) 1.0 (ref) 1.018 (0.951, 1.089) 0.925 (0.837, 1.022) | 2609 9182 4261 1413 | 798 (30.6) vs. 1811 (69.4) 2747 (29.9) vs. 6435 (70.1) 734 (17.2) vs. 3527 (82.8) 549 (38.9) vs. 864 (61.1) | 1.005 (0.937, 1.078) 1.0 (ref) 0.615 (0.567, 0.668) 1.196 (1.106, 1.295) |
| **CHA_2_DS_2_-VASc score**   Low: 1 for women   Moderate: 1 for men or 2 for women   High: ≥2 for men or ≥3 for women | 148  1958  9661 | 70 (47.3) vs. 78 (52.7)  762 (38.9) vs. 1196 (61.1) 3941 (40.8) vs. 5720 (59.2) | 1.0 (ref) 0.854 (0.739, 0.987) 0.847 (0.736, 0.975) | 241 2988 14,236 | 73 (30.3) vs. 168 (69.7) 759 (25.4) vs. 2229 (74.6) 3996 (28.1) vs. 10,240 (71.9) | 1.0 (ref) 0.847 (0.698, 1.029) 0.868 (0.718, 1.048) |
| **HAS-BLED (imputed) risk score**   Low: <3   High: ≥3 | 10801  966 | 4372 (40.5) vs. 6429 (59.5)  401 (41.5) vs. 565 (58.5) | 1.0 (ref) 0.960 (0.890, 1.034) | 16,022 1443 | 4403 (27.5) vs. 11,619 (72.5) 425 (29.5) vs. 1018 (70.5) | 1.0 (ref) 0.976 (0.898, 1.062) |
| **Type of AF**  Paroxysmal   Persistent   Permanent | 5923  4405  1439 | 2224 (37.5) vs. 3699 (62.5)  1929 (43.8) vs. 2476 (56.2)  620 (43.1) vs. 819 (56.9) | 0.962 (0.902, 1.025) 1.076 (1.010, 1.145) 1.0 (ref) | 9319 6301 1845 | 2179 (23.4) vs. 7140 (76.6) 1968 (31.2) vs. 4333 (68.8) 681 (36.9) vs. 1164 (63.1) | 0.750 (0.701, 0.803) 0.930 (0.871, 0.993) 1.0 (ref) |
| **Categorization of AF**  Symptomatic   Minimally symptomatic   Asymptomatic | 3407  4560  3800 | 1349 (39.6) vs. 2058 (60.4)  1897 (41.6) vs. 2663 (58.4)  1527 (40.2) vs. 2273 (59.8) | 0.992 (0.942, 1.046) 1.010 (0.963, 1.059) 1.0 (ref) | 5405 5823 6237 | 1611 (29.8) vs. 3794 (70.2) 1638 (28.1) vs. 4185 (71.9) 1579 (25.3) vs. 4658 (74.7) | 1.189 (1.122, 1.259) 1.138 (1.074, 1.205) 1.0 (ref) |
| **AF cardioversion**  Yes   No | 1976  9791 | 595 (30.1) vs. 1381 (69.9)  4178 (42.7) vs. 5613 (57.3) | 0.695 (0.648, 0.746) 1.0 (ref) | 3210 14,255 | 695 (21.7) vs. 2515 (78.3) 4133 (29.0) vs. 10,122 (71.0) | 0.772 (0.719, 0.829) 1.0 (ref) |
| **Creatinine clearance, mL/min**  <30   30 to <50   50 to <80   ≥80 | 353  1835  4620  4960 | 215 (60.9) vs. 138 (39.1)  806 (43.9) vs. 1029 (56.1)  1902 (41.2) vs. 2718 (58.8)  1850 (37.3) vs. 3110 (62.7) | 1.353 (1.257, 1.457) 1.092 (1.013, 1.176) 1.066 (1.009, 1.126) 1.0 (ref) | 505 2556 6874 7530 | 242 (47.9) vs. 264 (52.3)  799 (31.3) vs. 1757 (68.7) 1878 (27.3) vs. 4996 (72.7) 1909 (25.4) vs. 5621 (74.6) | 1.417 (1.287, 1.561) 1.137 (1.050, 1.231) 1.025 (0.963, 1.091) 1.0 (ref) |
| **Cancer**  Yes   No | 1181 10,586 | 458 (38.8) vs. 723 (61.2)  4315 (40.8) vs. 6271 (59.2) | 1.035 (0.964, 1.112) 1.0 (ref) | 1821 15,644 | 484 (26.6) vs. 1336 (73.4) 4344 (27.8) vs. 11301 (72.2) | 0.992 (0.923, 1.066) 1.0 (ref) |
| **Chronic gastrointestinal disease**  Yes  No | 1608 10,159 | 602 (37.4) vs. 1006 (62.6)  4171 (41.1) vs. 5988 (58.9) | 0.954 (0.895, 1.016) 1.0 (ref) | 2343 15,122 | 570 (24.3) vs. 1773 (75.7) 4258 (28.2) vs. 10,864 (71.8) | 0.958 (0.891, 1.029) 1.0 (ref) |
| **Hyperlipidemia**  Yes  No | 5024  6743 | 1925 (38.3) vs. 3099 (61.7)  2848 (42.2) vs. 3895 (57.8) | 0.973 (0.931, 1.017) 1.0 (ref) | 7251 10,214 | 1892 (26.1) vs. 5359 (73.9) 2936 (28.7) vs. 7278 (71.3) | 0.955 (0.908, 1.005) 1.0 (ref) |
| **Coronary artery disease**  Yes  No | 2229  9538 | 896 (40.2) vs. 1332 (59.8)  3877 (40.6) vs. 5662 (59.4) | 1.005 (0.950, 1.064) 1.0 (ref) | 3099 14,366 | 944 (30.5) vs. 2154 (69.5) 3884 (27.0) vs. 10,483 (73.0) | 1.111 (1.045, 1.181) 1.0 (ref) |
| **Smoking status**  Nonsmoker   Current smoker  Past smoker | 7014  1011  3742 | 2787 (39.7) vs. 4227 (60.3)  451 (44.6) vs. 559 (55.3)  1535 (41.0) vs. 2208 (59.0) | 1.0 (ref) 1.052 (0.990, 1.118) 1.054 (1.007, 1.102) | 10,237 1598 5630 | 2840 (27.7) vs. 7397 (72.3) 456 (28.5) vs. 1143 (71.5) 1532 (27.2) vs. 4097 (72.8) | 1.0 (ref) 1.071 (0.988, 1.160) 1.061 (1.007, 1.118) |
| **AP drug use**^§^  Yes   No | 2098  9669 | 929 (44.3) vs. 1169 (55.7)  3844 (39.8) vs. 5825 (60.2) | 1.151 (1.093, 1.211) 1.0 (ref) | 3053 14,412 | 888 (29.1) vs. 2165 (70.9) 3940 (27.3) vs. 10,472 (72.7) | 1.194 (1.117, 1.277) 1.0 (ref) |
| **Physician specialty**  GP/PCP/geriatrician   Cardiologist   Neurologist   Internist   Other | 387  10,280 351 355 394 | 155 (40.1) vs. 232 (59.9)  4173 (40.6) vs. 6107 (59.4)  68 (19.4) vs. 283 (80.6)  141 (39.7) vs. 214 (60.3)  235 (59.6) vs. 158 (40.1) | 0.971 (0.861, 1.095) 1.0 (ref) 0.417 (0.337, 0.518) 0.988 (0.872, 1.119) 1.124 (1.037, 1.217) | 802 14,780 453 738 692 | 226 (28.2) vs. 576 (71.8) 3940 (26.7) vs. 10,840 (73.3) 70 (15.5) vs. 383 (84.5) 324 (43.9) vs. 414 (56.1) 268 (38.7) vs. 424 (61.3) | 0.894 (0.796, 1.005) 1.0 (ref) 0.555 (0.446, 0.690) 1.457 (1.351, 1.571) 1.296 (1.179, 1.425) |
| **Medical treatment reimbursement**  Self-pay/no coverage  Not self-pay | 767  11,000 | 320 (41.7) vs. 447 (58.3)  4453 (40.5) vs. 6547 (59.5) | 1.001 (0.921, 1.088) 1.0 (ref) | 897 16,568 | 236 (26.3) vs. 661 (73.7) 4592 (27.7) vs. 11,976 (72.3) | 0.813 (0.722, 0.916) 1.0 (ref) |
| **Type of site**  Specialist office  Community hospital   University hospital   Other^\|\|^ | 3514  3441  3702  1110 | 1076 (30.6) vs. 2438 (69.4)  1212 (35.2) vs. 2229 (64.8)  2125 (57.4) vs. 1577 (42.6)  360 (32.4) vs. 750 (67.6) | 0.851 (0.790, 0.916) 1.0 (ref) 1.591 (1.509, 1.678) 0.892 (0.809, 0.985) | 5197 5410 5349 1509 | 1107 (21.3) vs. 4090 (78.7) 1243 (23.0) vs. 4167 (77.0) 1947 (36.4) vs. 3402 (63.6) 531 (35.2) vs. 978 (64.8) | 1.130 (1.047, 1.219) 1.0 (ref) 1.569 (1.478, 1.666) 1.458 (1.332, 1.596) |

AF, atrial fibrillation; AP, antiplatelets; BMI, body mass index; CHA_2_DS_2_-VASc, congestive heart failure, hypertension, age ≥75 years, diabetes, stroke/transient ischemic attack/systemic embolism, vascular disease, age from 65–74 years, sex category (female); CI, confidence interval; F, female; GP, general practitioner; HAS-BLED, hypertension, abnormal renal/liver function, stroke, bleeding history or predisposition, labile international normalized ratio, elderly (>65 years), drugs or alcohol concomitantly; M, male; NOAC, novel/nonvitamin K oral anticoagulants; PCP, primary care physician; ref, reference; VKA, vitamin K antagonist.

^*^Excluding patients from Africa/the Middle East.

^†^The data of patients in combinations of oral anticoagulants treatment group is not considered. NOAC includes dabigatran, rivaroxaban, and apixaban.

^‡^Confidence intervals were calculated based on the likelihood method.

^§^AP use was defined as use at the baseline visit.

^||^GP/primary care, outpatient healthcare centre, anticoagulation clinics, and other.

**S6 Table.** **Factors associated with prescription of VKA versus NOAC using multiple imputation – Model 2**.

| **Factor** | **Multivariate analysis relative risk estimates from the log-binomial regression analysis** | | | | | |
| --- | --- | --- | --- | --- | --- | --- |
|  | **Phase II*** | | | **Phase III** | | |
|  | **Total N** | **VKA vs. NOAC**^†^**, n (%)** | **Relative proportion  (95% CI)**^‡^ | **Total N** | **VKA vs. NOAC**^†^**, n (%)** | **Relative proportion  (95% CI)**^‡^ |
| **BMI, kg/m^2^**  <18.5   18.5 to <25   25 to <30  30 to <35   ≥35 | 159  2933 4589 2485 1601 | 65 (40.9) vs. 95 (59.7)  1248 (42.6) vs. 1685 (57.4)  1869 (40.7) vs. 2720 (59.3)  971 (39.1) vs. 1514 (60.9) 620 (38.7) vs.981 (61.3) | 0.956 (0.789, 1.158) 1.0 (ref) 1.049 (0.998, 1.103) 1.057 (0.994, 1.125) 1.094 (1.021, 1.173) | 212 4521 6667 3614 2451 | 60 (28.3) vs. 152 (71.7)  1293 (28.6) vs. 3228 (71.4)  1878 (28.2) vs. 4789 (71.8)  967 (26.8) vs. 2647 (73.2)  630 (25.7) vs. 1822 (74.3) | 1.007 (0.810, 1.251) 1.0 (ref) 1.029 (0.971, 1.090) 1.050 (0.976, 1.130) 1.028 (0.946, 1.117) |
| **Region**  Asia   Europe   North America   Latin America | 1696 6624 2667 780 | 846 (49.9) vs. 850 (50.1) 2763 (41.7) vs. 3861 (58.3)  897 (33.6) vs. 1770 (66.4) 267 (34.2) vs. 513 (65.8) | 1.141 (1.079, 1.207) 1.0 (ref) 0.988 (0.922, 1.059) 0.933 (0.844, 1.031) | 2609 9182 4261 1413 | 798 (30.6) vs. 1811 (69.4)  2747 (29.9) vs. 6435 (70.1) 734 (17.2) vs. 3527 (82.8) 549 (38.9) vs. 864 (61.1) | 0.992 (0.923, 1.065) 1.0 (ref) 0.622 (0.573, 0.676) 1.126 (1.037, 1.223) |
| **Congestive heart failure/LV dysfunction**  Yes  No | 2866 8901 | 1226 (42.8) vs. 1639 (57.2) 3547 (39.8) vs. 5355 (60.2) | 0.989 (0.943, 1.036) 1.0 (ref) | 3892 13,574 | 1394 (35.8) vs. 2498 (64.2) 3434 (25.3) vs. 10,139 (74.7) | 1.192 (1.129, 1.259) 1.0 (ref) |
| **History of hypertension**  Yes  No | 8945 2822 | 3527 (39.4) vs. 5418 (60.6) 1246 (44.2) vs. 1576 (55.8) | 0.925 (0.883, 0.968) 1.0 (ref) | 13,308 4157 | 3649 (27.4) vs. 9659 (72.6) 1179 (28.4) vs. 2978 (71.6) | 0.965 (0.913, 1.020) 1.0 (ref) |
| **Abnormal kidney function**  Yes  No | 154 11613 | 117 (76.0) vs. 37 (24.0)  4656 (40.1) vs. 6957 (59.9) | 1.160 (1.047, 1.286) 1.0 (ref) | 283 17,182 | 174 (61.5) vs. 109 (38.5) 4654 (27.1) vs. 12,529 (72.9) | 1.287 (1.145, 1.448) 1.0 (ref) |
| **Diabetes mellitus**  Yes  No | 2713 9054 | 1176 (43.3) vs. 1537 (56.7) 3597 (39.7) vs. 5457 (60.3) | 1.060 (1.013, 1.108) 1.0 (ref) | 4160 13,305 | 1229 (29.5) vs. 2931 (70.5) 3599 (27.0) vs. 9706 (73.0) | 1.070 (1.016, 1.126) 1.0 (ref) |
| **Stroke/TIA/systemic embolism**  Yes  No | 557 11,210 | 223 (40.0) vs. 334 (60.0)  4550 (40.6) vs. 6660 (59.4) | 0.993 (0.902, 1.094) 1.0 (ref) | 841 16,624 | 200 (23.8) vs. 642 (76.3) 4628 (27.8) vs. 11,995 (72.2) | 0.847 (0.750, 0.956) 1.0 (ref) |
| **Vascular disease**^§^  Yes  No | 1546 10,221 | 711 (46.0) vs. 835 (54.0)  4062 (39.7) vs. 6159 (60.3) | 1.120 (1.056, 1.188) 1.0 (ref) | 2142 15,323 | 712 (33.2) vs. 1430 (66.8) 4116 (26.9) vs. 11,207 (73.1) | 1.080 (0.998, 1.169) 1.0 (ref) |
| **Age**  <65   65 to <75  ≥75 | 2864 4070 4833 | 1128 (39.4) vs. 1736 (60.6) 1597 (39.2) vs. 2473 (60.8) 2048 (42.4) vs. 2785 (57.6) | 1.013 (0.952, 1.077) 0.990 (0.941, 1.042) 1.0 (ref) | 4060 6480 6925 | 1158 (28.5) vs. 2902 (71.5) 1721 (26.6) vs. 4759 (73.4) 1949 (28.1) vs. 4976 (71.9) | 1.090 (1.017, 1.168) 1.010 (0.952, 1.071) 1.0 (ref) |
| **Sex**  Male  Female | 6447 5320 | 2555 (39.6) vs. 3892 (60.4)  2218 (41.7) vs. 3102 (58.3) | 1.0 (ref) 1.080 (1.035, 1.127) | 9614 7851 | 2681 (27.9) vs. 6933 (72.1) 2147 (27.3) vs. 5704 (72.7) | 1.0 (ref) 1.029 (0.979, 1.082) |
| **Hepatic disease**  Yes  No | 184 11,583 | 74 (40.2) vs. 109 (59.2)  4699 (40.6) vs. 6885 (59.4) | 0.969 (0.817, 1.149) 1.0 (ref) | 246 17,219 | 67 (27.2) vs. 179 (72.8) 4761 (27.6) vs. 12,458 (72.4) | 1.000 (0.817, 1.223) 1.0 (ref) |
| **Prior bleeding**  Yes  No | 651 10,875 | 282 (43.3) vs. 369 (56.7)  4418 (40.6) vs. 6457 (59.4) | 1.072 (0.990, 1.160) 1.0 (ref) | 862 16,603 | 248 (28.8) vs. 614 (71.2) 4580 (27.6) vs. 12,023 (72.4) | 1.069 (0.964, 1.185) 1.0 (ref) |
| **Alcohol abuse**  Yes  No | 913 10,854 | 367 (40.2) vs. 546 (59.8)  4406 (40.6) vs. 6448 (59.4) | 1.018 (0.937, 1.106) 1.0 (ref) | 1331 16,134 | 326 (24.5) vs. 1006 (75.6) 4502 (27.9) vs. 11,631 (72.1) | 0.936 (0.846, 1.037) 1.0 (ref) |
| **Type of AF**  Paroxysmal   Persistent   Permanent | 5923 4405 1439 | 2224 (37.5) vs. 3699 (62.5) 1929 (43.8) vs. 2476 (56.2)  620 (43.1) vs. 819 (56.9) | 0.995 (0.938, 1.055) 1.113 (1.048, 1.182) 1.0 (ref) | 9319 6301 1845 | 2179 (23.4) vs. 7140 (76.6)  1968 (31.2) vs. 4333 (68.8)  681 (36.9) vs. 1164 (63.1) | 0.757 (0.708, 0.811) 0.897 (0.840, 0.958) 1.0 (ref) |
| **Categorization of AF**  Symptomatic   Minimally symptomatic   Asymptomatic | 3407 4560 3800 | 1349 (39.6) vs. 2058 (60.4) 1897 (41.6) vs. 2663 (58.4) 1527 (40.2) vs. 2273 (59.8) | 0.995 (0.943, 1.050) 1.015 (0.970, 1.062) 1.0 (ref) | 5405 5823 6237 | 1611 (29.8) vs. 3794 (70.2) 1638 (28.1) vs. 4185 (71.9) 1579 (25.3) vs. 4658 (74.7) | 1.123 (1.059, 1.191) 1.109 (1.047, 1.175) 1.0 (ref) |
| **AF cardioversion**  Yes   No | 1976 9791 | 595 (30.1) vs. 1381 (69.9)  4178 (42.7) vs. 5613 (57.3) | 0.694 (0.646, 0.745) 1.0 (ref) | 3210 14,255 | 695 (21.7) vs. 2515 (78.3) 4133 (29.0) vs. 10,122 (71.0) | 0.777 (0.723, 0.835) 1.0 (ref) |
| **Creatinine clearance, mL/min**  <30   30 to <50   50 to <80   ≥80 | 353 1835 4620 4960 | 215 (60.9) vs. 138 (39.1)  806 (43.9) vs. 1029 (56.1) 1902 (41.2) vs. 2718 (58.8) 1850 (37.3) vs. 3110 (62.7) | 1.213 (1.088, 1.353) 1.055 (0.973, 1.144) 1.050 (0.991, 1.113) 1.0 (ref) | 505 2556 6874 7530 | 242 (47.9) vs. 264 (52.3)  799 (31.3) vs. 1757 (68.7) 1878 (27.3) vs. 4996 (72.7)  1909 (25.4) vs. 5621 (74.6) | 1.180 (1.037, 1.343) 1.107 (1.012, 1.212) 1.026 (0.962, 1.093) 1.0 (ref) |
| **Cancer**  Yes   No | 1181 10,586 | 458 (38.8) vs. 723 (61.2)  4315 (40.8) vs. 6271 (59.2) | 1.027 (0.957, 1.102) 1.0 (ref) | 1821 15,644 | 484 (26.6) vs. 1336 (73.4) 4344 (27.8) vs. 11,301 (72.2) | 1.039 (0.964, 1.119) 1.0 (ref) |
| **Hyperlipidemia**  Yes  No | 5024 6743 | 1925 (38.3) vs. 3099 (61.7) 2848 (42.2) vs. 3895 (57.8) | 0.968 (0.925, 1.013) 1.0 (ref) | 7251 10,214 | 1892 (26.1) vs. 5359 (73.9) 2936 (28.7) vs. 7278 (71.3) | 0.966 (0.917, 1.018) 1.0 (ref) |
| **Coronary artery disease**  Yes  No | 2229 9538 | 896 (40.2) vs. 1332 (59.8) 3877 (40.6) vs. 5662 (59.4) | 0.969 (0.909, 1.033) 1.0 (ref) | 3099 14,366 | 944 (30.5) vs. 2154 (69.5) 3884 (27.0) vs. 10,483 (73.0) | 1.042 (0.966, 1.123) 1.0 (ref) |
| **Chronic gastrointestinal disease**  Yes  No | 1608 10,159 | 602 (37.4) vs. 1006 (62.6)  4171 (41.1) vs. 5988 (58.9) | 0.944 (0.884, 1.007) 1.0 (ref) | 2343 15,122 | 570 (24.3) vs. 1773 (75.7) 4258 (28.2) vs. 10,864 (71.8) | 0.945 (0.879, 1.016) 1.0 (ref) |
| **Smoking status**  Nonsmoker   Current smoker  Past smoker | 7014 1011 3742 | 2787 (39.7) vs. 4227 (60.3)  451 (44.6) vs. 559 (55.3)  1535 (41.0) vs. 2208 (59.0) | 1.0 (ref) 1.083 (1.012, 1.160) 1.069 (1.023, 1.117) | 10,237 1598 5630 | 2840 (27.7) vs. 7397 (72.3)  456 (28.5) vs. 1143 (71.5)  1532 (27.2) vs. 4097 (72.8) | 1.0 (ref) 1.056 (0.970, 1.149) 1.036 (0.982, 1.094) |
| **AP drug use**^\|\|^  Yes   No | 2098 9669 | 929 (44.3) vs. 1169 (55.7)  3844 (39.8) vs. 5825 (60.2) | 1.106 (1.056, 1.159) 1.0 (ref) | 3053 14,412 | 888 (29.1) vs. 2165 (70.9) 3940 (27.3) vs. 10,472 (72.7) | 1.153 (1.086, 1.224) 1.0 (ref) |
| **Physician specialty**  GP/PCP/geriatrician   Cardiologist   Neurologist   Internist   Other | 387 10,280 351 355 394 | 155 (40.1) vs. 232 (59.9)  4173 (40.6) vs. 6107 (59.4) 68 (19.4) vs. 283 (80.6) 141 (39.7) vs. 214 (60.3)  235 (59.6) vs. 158 (40.1) | 0.944 (0.834, 1.068) 1.0 (ref) 0.412 (0.332, 0.512) 0.997 (0.880, 1.130) 1.124 (1.035, 1.220) | 802 14,780 453 738 692 | 226 (28.2) vs. 576 (71.8)  3940 (26.7) vs. 10,840 (73.3)  70 (15.5) vs. 383 (84.5) 324 (43.9) vs. 414 (56.1)  268 (38.7) vs. 424 (61.3) | 0.921 (0.820, 1.035) 1.0 (ref) 0.566 (0.456, 0.704) 1.378 (1.276, 1.487) 1.362 (1.238, 1.497) |
| **Medical treatment reimbursement**  Self-pay/no coverage  Not self-pay | 767 11,000 | 320 (41.7) vs. 447 (58.3)  4453 (40.5) vs. 6547 (59.5) | 0.971 (0.905, 1.043) 1.0 (ref) | 897 16,568 | 236 (26.3) vs. 661 (73.7)  4592 (27.7) vs. 11,976 (72.3) | 0.830 (0.736, 0.936) 1.0 (ref) |
| **Type of site**  Specialist office  Community hospital   University hospital   Other^¶^ | 3514 3441 3702 1110 | 1076 (30.6) vs. 2438 (69.4)  1212 (35.2) vs. 2229 (64.8)  2125 (57.4) vs. 1577 (42.6)  360 (32.4) vs. 750 (67.6) | 0.874 (0.811, 0.942) 1.0 (ref) 1.570 (1.488, 1.656) 0.893 (0.809, 0.986) | 5197 5410 5349 1509 | 1107 (21.3) vs. 4090 (78.7)  1243 (23.0) vs. 4167 (77.0)  1947 (36.4) vs. 3402 (63.6)  531 (35.2) vs. 978 (64.8) | 1.125 (1.042, 1.214) 1.0 (ref) 1.551 (1.461, 1.647) 1.400 (1.276, 1.537) |

AF, atrial fibrillation; AP, antiplatelets; BMI, body mass index; CI, confidence interval; GP, general practitioner; LV, left ventricle; NOAC, novel/nonvitamin K oral anticoagulants; OAC, oral anticoagulation; PCP, primary care physician; ref, reference; TIA, transient ischemic attack; VKA, vitamin K antagonist.

^*^Excluding patients from Africa/the Middle East.

^†^The data of patients in combinations of oral anticoagulants treatment group are not considered. NOAC includes dabigatran, rivaroxaban, and apixaban.

^‡^CIs were calculated based on the likelihood method.

^§^Prior myocardial infarction, peripheral artery disease, complex aortic plaque.

^||^AP use was defined as use at the baseline visit.

^¶^GP/primary care, outpatient healthcare centre, anticoagulation clinics, and other.

**S7 Table. Factors associated with prescription of AP versus no OAC using multiple imputation – Model 1.**

| **Factor** | **Multivariate analysis relative risk estimates from the log-binomial regression analysis** | | | | | |
| --- | --- | --- | --- | --- | --- | --- |
|  | **Phase II*** | | | **Phase III** | | |
|  | **Total N** | **AP vs. no OAC**^†^**, n (%)** | **Relative proportion (95% CI)**^‡^ | **Total N** | **AP vs. no OAC**^†^**, n (%)** | **Relative proportion  (95% CI)**^‡^ |
| **BMI, kg/m^2^**  <18.5   18.5 to <25   25 to <30  30 to <35   ≥35 | 60  1094 1102 438 243 | 34 (56.7) vs. 27 (45.0)  635 (58.0) vs. 459 (42.0)  674 (61.2) vs. 428 (38.8)  266 (60.7) vs. 172 (39.3)  167 (68.7) vs. 76 (31.3) | 1.010 (0.806, 1.265) 1.0 (ref) 1.000 (0.961, 1.041) 0.985 (0.913, 1.064) 1.036 (0.976, 1.099) | 72  1428 1385 570 320 | 44 (61.1) vs. 28 (38.9)  866 (60.6) vs. 562 (39.4)  876 (63.2) vs. 509 (36.8)  370 (64.9) vs. 200 (35.1) 216 (67.5) vs. 104 (32.5) | 1.041 (0.904, 1.198) 1.0 (ref) 1.022 (0.985, 1.062) 1.048 (0.999, 1.100) 1.001 (0.957, 1.047) |
| **Region**  Asia   Europe   North America   Latin America | 1374 708  724  131 | 770 (56.0) vs. 604 (44.0)  436 (61.6) vs. 272 (38.4)  477 (65.9) vs. 247 (34.1)  93 (71.0) vs. 38 (29.0) | 0.922 (0.870, 0.977) 1.0 (ref) 1.006 (0.943, 1.074) 1.119 (1.035, 1.211) | 1630  1097 836 213 | 1032 (63.3) vs. 598 (36.7)  587 (53.5) vs. 510 (46.5) 619 (74.0) vs. 217 (26.0) 135 (63.4) vs. 78 (36.6) | 1.083 (1.011, 1.159) 1.0 (ref) 1.159 (1.082, 1.241) 1.102 (1.015, 1.196) |
| **CHA_2_DS_2_-VASc score**  Low: 1 for women   Moderate: 1 for men or 2 for women   High: ≥2 for men or ≥3 for women | 176  801 1960 | 97 (55.1) vs. 79 (44.9)  483 (60.3) vs. 318 (39.7) 1196 (61.0) vs. 764 (39.0) | 1.0 (ref) 1.084 (0.940, 1.250) 0.942 (0.814, 1.090) | 247 979 2550 | 118 (47.8) vs. 129 (52.2) 594 (60.7) vs. 385 (39.3) 1661 (65.1) vs. 889 (34.9) | 1.0 (ref) 1.194 (1.043, 1.367) 1.164 (1.016, 1.333) |
| **HAS-BLED (imputed) risk score**  Low: <3   High: ≥3 | 2388 549 | 1304 (54.6) vs. 1085 (45.4)  472 (86.0) vs. 76 (13.8) | 1.0 (ref) 1.517 (1.435, 1.604) | 3010 766 | 1715 (57.0) vs. 1295 (43.0) 658 (85.9) vs. 108 (14.1) | 1.0 (ref) 1.292 (1.229, 1.359) |
| **Type of AF**  Paroxysmal   Persistent   Permanent | 1944  859  134 | 1200 (61.7) vs. 744 (38.3)  490 (57.0) vs. 369 (43.0)  86 (64.2) vs. 48 (35.8) | 1.016 (0.924, 1.117) 0.981 (0.880, 1.093) 1.0 (ref) | 2653 948 175 | 1716 (64.7) vs. 937 (35.3) 553 (58.3) vs. 395 (41.7) 104 (59.4) vs. 71 (40.6) | 1.055 (0.943, 1.181) 1.001 (0.890, 1.125) 1.0 (ref) |
| **Categorization of AF**  Symptomatic   Minimally symptomatic   Asymptomatic | 748  1264  925 | 490 (65.5) vs. 258 (34.5) 742 (58.7) vs. 522 (41.3)  544 (58.8) vs. 381 (41.2) | 1.039 (0.985, 1.097) 1.011 (0.953, 1.073) 1.0 (ref) | 1183 1393 1200 | 745 (63.0) vs. 438 (37.0) 865 (62.1) vs. 528 (37.9) 763 (63.6) vs. 437 (36.4) | 0.989 (0.950, 1.029) 0.959 (0.923, 0.997) 1.0 (ref) |
| **AF cardioversion**  Yes   No | 412 2525 | 256 (62.1) vs. 156 (37.9)  1520 (60.2) vs. 1005 (39.8) | 1.009 (0.951, 1.071) 1.0 (ref) | 662 3114 | 431 (65.1) vs. 231 (34.9) 1942 (62.4) vs. 1172 (37.6) | 1.010 (0.974, 1.047) 1.0 (ref) |
| **Creatinine clearance, mL/min**  <30   30 to <50   50 to <80   ≥80 | 117  472 1112 1236 | 66 (56.4) vs. 52 (44.4)  308 (65.3) vs. 164 (34.7) 653 (58.7) vs. 459 (41.3)  749 (60.6) vs. 486 (39.3) | 0.833 (0.718, 0.966) 0.993 (0.927, 1.065) 0.958 (0.904, 1.015) 1.0 (ref) | 136 529 1384 1726 | 96 (70.6) vs. 40 (29.4)  338 (63.9) vs. 191 (36.1) 857 (61.9) vs. 527 (38.1) 1082 (62.7) vs. 645 (37.4) | 1.045 (0.993, 1.099) 1.037 (0.981, 1.096) 1.015 (0.976, 1.056) 1.0 (ref) |
| **Cancer**  Yes   No | 237 2700 | 136 (57.4) vs. 100 (42.2)  1640 (60.7) vs. 1061 (39.3) | 0.977 (0.902, 1.057) 1.0 (ref) | 321 3455 | 184 (57.3) vs. 136 (42.4) 2189 (63.4) vs. 1267 (36.7) | 0.954 (0.873, 1.041) 1.0 (ref) |
| **Chronic gastrointestinal disease**  Yes  No | 388  2549 | 235 (60.6) vs. 153 (39.4)  1541 (60.5) vs. 1008 (39.5) | 0.970 (0.914, 1.029) 1.0 (ref) | 513 3263 | 321 (62.6) vs. 193 (37.6) 2052 (62.9) vs. 1210 (37.1) | 0.989 (0.955, 1.025) 1.0 (ref) |
| **Hyperlipidemia**  Yes  No | 942 1995 | 642 (68.2) vs. 300 (31.8) 1134 (56.8) vs. 861 (43.2) | 1.058 (1.000, 1.119) 1.0 (ref) | 1202 2574 | 856 (71.2) vs. 347 (28.9) 1517 (58.9) vs. 1056 (41.0) | 1.010 (0.974, 1.047) 1.0 (ref) |
| **Coronary artery disease**  Yes  No | 732 2205 | 530 (72.4) vs. 202 (27.6)  1246 (56.5) vs. 959 (43.5) | 1.138 (1.077, 1.203) 1.0 (ref) | 927 2849 | 769 (83.0) vs. 157 (16.9) 1604 (56.3) vs. 1246 (43.7) | 1.233 (1.169, 1.301) 1.0 (ref) |
| **Smoking status**  Nonsmoker   Current smoker  Past smoker | 1788 405 744 | 1051 (58.8) vs. 738 (41.3)  244 (60.2) vs. 161 (39.8)  482 (64.8) vs. 262 (35.2) | 1.0 (ref) 0.983 (0.909, 1.064) 0.994 (0.952, 1.039) | 2301 491 984 | 1418 (61.6) vs. 883 (38.4) 327 (66.6) vs. 164 (33.4) 628 (63.8) vs. 356 (36.2) | 1.0 (ref) 1.053 (1.019, 1.088) 1.012 (0.980, 1.044) |
| **AP drug use**^§^  Yes   No | 1759 1178 | 1736 (98.7) vs. 23 (1.3)  40 (3.4) vs. 1138 (96.6) | NA NA | 2372 1404 | 2270 (95.7) vs. 102 (4.3) 103 (7.3) vs. 1301 (92.7) | NA NA |
| **Physician specialty**  GP/PCP/geriatrician   Cardiologist   Neurologist   Internist   Other | 114  2638  51 70 64 | 82 (71.9) vs. 32 (28.1)  1589 (60.2) vs. 1049 (39.8) 41 (80.4) vs. 10 (19.6)  36 (51.4) vs. 34 (48.6)  28 (43.8) vs. 36 (56.3) | 1.028 (0.973, 1.087) 1.0 (ref) 1.049 (0.950, 1.158) 0.853 (0.682, 1.067) 0.781 (0.592, 1.030) | 256 3277 71 82 90 | 140 (54.7) vs. 116 (45.3) 2108 (64.3) vs. 1169 (35.7) 37 (52.1) vs. 34 (47.9)  49 (59.8) vs. 33 (40.2)  39 (43.3) vs. 51 (56.7) | 0.897 (0.803, 1.002) 1.0 (ref) 0.808 (0.648, 1.007) 0.934 (0.787, 1.107) 0.766 (0.604, 0.972) |
| **Medical treatment reimbursement**  Self-pay/no coverage  Not self-pay | 274 2663 | 199 (72.6) vs. 76 (27.7) 1577 (59.2) vs. 1086 (40.8) | 1.123 (1.056, 1.193) 1.0 (ref) | 180 3596 | 118 (65.6) vs. 62 (34.4) 2255 (62.7) vs. 1341 (37.3) | 1.060 (1.007, 1.115) 1.0 (ref) |
| **Type of site**  Specialist office  Community hospital   University hospital   Other^\|\|^ | 911 531 1198 297 | 598 (65.6) vs. 313 (34.4) 339 (63.8) vs. 192 (36.2)  673 (56.2) vs. 525 (43.8) 166 (55.9) vs. 131 (44.1) | 0.995 (0.927, 1.068) 1.0 (ref) 0.996 (0.933, 1.062) 0.919 (0.835, 1.012) | 1019 842 1407 508 | 719 (70.6) vs. 300 (29.4) 502 (59.6) vs. 340 (40.4) 804 (57.1) vs. 603 (42.9) 348 (68.5) vs. 160 (31.5) | 0.985 (0.950, 1.021) 1.0 (ref) 0.919 (0.868, 0.972) 1.001 (0.953, 1.051) |

AF, atrial fibrillation; AP, antiplatelets; BMI, body mass index; CHA_2_DS_2_-VASc, congestive heart failure, hypertension, age ≥75 years, diabetes, stroke/transient ischemic attack/systemic embolism, vascular disease, age from 65–74 years, sex category (female); CI, confidence interval; F, female; GP, general practitioner; HAS-BLED, hypertension, abnormal renal/liver function, stroke, bleeding history or predisposition, labile international normalized ratio, elderly (>65 years), drugs or alcohol concomitantly; M, male; NA, not applicable; OAC, oral anticoagulant; PCP, primary care physician; ref, reference; VKA, vitamin K antagonist.

^*^Excluding patients from Africa/the Middle East.

^†^The data of patients in combinations of oral anticoagulants treatment group are not considered. OAC use includes dabigatran, VKA, rivaroxaban, and apixaban. AP includes AP without OAC.

^‡^CIs were calculated based on the likelihood method.

^§^AP use was defined as use at the baseline visit.

^||^GP/primary care, outpatient healthcare centre, anticoagulation clinics, and other.

**S8 Table.** **Factors associated with prescription of AP versus no OAC using multiple imputation – Model 2**

| **Factor** | **Multivariate analysis relative risk estimates from the log-binomial regression analysis** | | | | | |
| --- | --- | --- | --- | --- | --- | --- |
|  | **Phase II*** | | | **Phase III** | | |
|  | **Total N** | **AP vs. no OAC**^†^**, n (%)** | **Relative proportion  (95% CI)**^‡^ | **Total N** | **AP vs. No OAC**^†^**, n (%)** | **Relative proportion  (95% CI)**^‡^ |
| **BMI, kg/m^2^**  <18.5   18.5 to <25   25 to <30  30 to <35   ≥35 | 60 1094 1102 438 243 | 34 (56.7) vs. 27 (45.0) 635 (58.0) vs. 459 (42.0)  674 (61.2) vs. 428 (38.8)  266 (60.7) vs. 172 (39.3)  167 (68.7) vs. 76 (31.3) | 0.951 (0.753, 1.199) 1.0 (ref) 1.029 (0.961, 1.101) 0.999 (0.910, 1.098) 1.048 (0.943, 1.164) | 72 1428 1385 570 320 | 44 (61.1) vs. 28 (38.9) 866 (60.6) vs. 562 (39.4)  876 (63.2) vs. 509 (36.8) 370 (64.9) vs. 200 (35.1)  216 (67.5) vs. 104 (32.5) | 0.982 (0.832, 1.160) 1.0 (ref) 1.017 (0.979, 1.057) 0.999 (0.938, 1.063) 0.996 (0.910, 1.090) |
| **Region**  Asia   Europe   North America   Latin America | 1374 708  724  131 | 770 (56.0) vs. 604 (44.0)  436 (61.6) vs. 272 (38.4) 477 (65.9) vs. 247 (34.1) 93 (71.0) vs. 38 (29.0) | 0.934 (0.858, 1.015) 1.0 (ref) 1.000 (0.910, 1.098) 1.035 (0.893, 1.199) | 1630 1097 836 213 | 1032 (63.3) vs. 598 (36.7)  587 (53.5) vs. 510 (46.5)  619 (74.0) vs. 217 (26.0) 135 (63.4) vs. 78 (36.6) | 1.117 (1.035, 1.205) 1.0 (ref) 1.156 (1.063, 1.257) 1.085 (0.976, 1.206) |
| **Congestive heart failure/LV dysfunction**  Yes  No | 665 2272 | 439 (66.0) vs. 226 (34.0) 1337 (58.8) vs. 935 (41.2) | 1.045 (0.978, 1.117) 1.0 (ref) | 762 3014 | 520 (68.2) vs. 242 (31.8)  1853 (61.5) vs. 1161 (38.5) | 1.008 (0.961, 1.058) 1.0 (ref) |
| **History of hypertension**  Yes  No | 2006 931 | 1217 (60.7) vs. 788 (39.3)  559 (60.0) vs. 373 (40.1) | 0.931 (0.873, 0.992) 1.0 (ref) | 2554 1222 | 1670 (65.4) vs. 884 (34.6)  703 (57.5) vs. 519 (42.5) | 1.027 (0.972, 1.086) 1.0 (ref) |
| **Abnormal kidney function**  Yes  No | 73 2864 | 45 (61.6) vs. 28 (38.4)  1731 (60.4) vs. 1133 (39.6) | 0.978 (0.792, 1.208) 1.0 (ref) | 112 3664 | 83 (74.1) vs. 29 (25.9)  2290 (62.5) vs. 1374 (37.5) | 1.038 (0.947, 1.138) 1.0 (ref) |
| **Diabetes mellitus**  Yes  No | 566 2371 | 373 (65.9) vs. 193 (34.1)  1403 (59.2) vs. 968 (40.8) | 1.033 (0.969, 1.102) 1.0 (ref) | 780 2996 | 531 (68.1) vs. 249 (31.9)  1842 (61.5) vs. 1154 (38.5) | 0.991 (0.946, 1.039) 1.0 (ref) |
| **Stroke/TIA/systemic embolism**  Yes  No | 89 2849 | 50 (56.2) vs. 38 (42.7)  1726 (60.6) vs. 1123 (39.4) | 0.930 (0.776, 1.115) 1.0 (ref) | 115 3661 | 75 (65.2) vs. 40 (34.8)  2298 (62.8) vs. 1363 (37.2) | 0.992 (0.898, 1.094) 1.0 (ref) |
| **Vascular disease**^§^  Yes  No | 423 2514 | 318 (75.2) vs. 105 (24.8)  1458 (58.0) vs. 1056 (42.0) | 1.048 (0.969, 1.132) 1.0 (ref) | 549 3227 | 462 (84.2) vs. 87 (15.8) 1911 (59.2) vs. 1316 (40.8) | 1.080 (1.020, 1.144) 1.0 (ref) |
| **Age**  <65   65 to <75  ≥75 | 1051 920 966 | 638 (60.7) vs. 413 (39.3)  553 (60.1) vs. 367 (39.9)  585 (60.6) vs. 381 (39.4) | 0.989 (0.911, 1.075) 0.978 (0.911, 1.049) 1.0 (ref) | 1354 1212 1210 | 842 (62.2) vs. 512 (37.8) 760 (62.7) vs. 452 (37.3) 771 (63.7) vs. 439 (36.3) | 1.010 (0.944, 1.081) 0.984 (0.930, 1.041) 1.0 (ref) |
| **Sex**  Male  Female | 1579 1358 | 970 (61.4) vs. 609 (38.6)  806 (59.4) vs. 552 (40.6) | 1.0 (ref) 0.995 (0.935, 1.059) | 2081 1695 | 1346 (64.7) vs. 735 (35.3)  1027 (60.6) vs. 668 (39.4) | 1.0 (ref) 0.980 (0.935, 1.027) |
| **Hepatic disease**  Yes  No | 64 2873 | 27 (42.2) vs. 37 (57.8) 1749 (60.9) vs. 1124 (39.1) | 0.715 (0.533, 0.960) 1.0 (ref) | 77 3699 | 51 (66.2) vs. 27 (35.1)  2322 (62.8) vs. 1376 (37.2) | 1.017 (0.882, 1.173) 1.0 (ref) |
| **Prior bleeding**  Yes  No | 188 2718 | 112 (59.6) vs. 76 (40.4) 1647 (60.6) vs. 1071 (39.4) | 0.945 (0.841, 1.063) 1.0 (ref) | 262 3514 | 149 (56.9) vs. 113 (43.1)  2224 (63.3) vs. 1290 (36.7) | 0.891 (0.806, 0.986) 1.0 (ref) |
| **Alcohol abuse**  Yes  No | 200 2737 | 129 (64.5) vs. 71 (35.5)  1647 (60.2) vs. 1090 (39.8) | 1.100 (0.998, 1.213) 1.0 (ref) | 241 3535 | 137 (56.8) vs. 104 (43.2)  2236 (63.3) vs. 1299 (36.7) | 0.997 (0.890, 1.117) 1.0 (ref) |
| **Type of AF**  Paroxysmal   Persistent   Permanent | 1944 859 134 | 1200 (61.7) vs. 744 (38.3) 490 (57.0) vs. 369 (43.0) 86 (64.2) vs. 48 (35.8) | 1.016 (0.888, 1.161) 0.939 (0.816, 1.080) 1.0 (ref) | 2653 948  175 | 1716 (64.7) vs. 937 (35.3)  553 (58.3) vs. 395 (41.7) 104 (59.4) vs. 71 (40.6) | 1.029 (0.911, 1.164) 0.998 (0.878, 1.133) 1.0 (ref) |
| **Categorization of AF**  Symptomatic   Minimally symptomatic   Asymptomatic | 748 1264 925 | 490 (65.5) vs. 258 (34.5) 742 (58.7) vs. 522 (41.3) 544 (58.8) vs. 381 (41.2) | 1.094 (1.017, 1.177) 1.015 (0.947, 1.088) 1.0 (ref) | 1183 1393 1200 | 745 (63.0) vs. 438 (37.0)  865 (62.1) vs. 528 (37.9)  763 (63.6) vs. 437 (36.4) | 1.017 (0.979, 1.057) 1.010 (0.962, 1.061) 1.0 (ref) |
| **AF cardioversion**  Yes   No | 412 2525 | 256 (62.1) vs. 156 (37.9)  1520 (60.2) vs. 1005 (39.8) | 0.987 (0.914, 1.065) 1.0 (ref) | 662 3114 | 431 (65.1) vs. 231 (34.9) 1942 (62.4) vs. 1172 (37.6) | 1.014 (0.960, 1.070) 1.0 (ref) |
| **Creatinine clearance, mL/min**  <30   30 to <50   50 to <80   ≥80 | 117 472 1112 1236 | 66 (56.4) vs. 52 (44.4) 308 (65.3) vs. 164 (34.7) 653 (58.7) vs. 459 (41.3) 749 (60.6) vs. 486 (39.3) | 0.897 (0.726, 1.109) 1.075 (0.978, 1.180) 0.986 (0.911, 1.066) 1.0 (ref) | 136 529 1384 1726 | 96 (70.6) vs. 40 (29.4)  338 (63.9) vs. 191 (36.1)  857 (61.9) vs. 527 (38.1)  1082 (62.7) vs. 645 (37.4) | 1.055 (0.940, 1.185) 1.012 (0.938, 1.091) 0.996 (0.939, 1.058) 1.0 (ref) |
| **Cancer**  Yes   No | 237 2700 | 136 (57.4) vs. 100 (42.2)  1640 (60.7) vs. 1061 (39.3) | 0.954 (0.852, 1.068) 1.0 (ref) | 321 3455 | 184 (57.3) vs. 136 (42.4)  2189 (63.4) vs. 1267 (36.7) | 0.907 (0.830, 0.990) 1.0 (ref) |
| **Hyperlipidemia**  Yes  No | 942 1995 | 642 (68.2) vs. 300 (31.8) 1134 (56.8) vs. 861 (43.2) | 1.104 (1.032, 1.182) 1.0 (ref) | 1202 2574 | 856 (71.2) vs. 347 (28.9)  1517 (58.9) vs. 1056 (41.0) | 1.051 (0.997, 1.108) 1.0 (ref) |
| **Coronary artery disease**  Yes  No | 732 2205 | 530 (72.4) vs. 202 (27.6)  1246 (56.5) vs. 959 (43.5) | 1.185 (1.101, 1.275) 1.0 (ref) | 927 2849 | 769 (83.0) vs. 157 (16.9)  1604 (56.3) vs. 1246 (43.7) | 1.321 (1.245, 1.403) 1.0 (ref) |
| **Chronic gastrointestinal disease**  Yes  No | 388 2549 | 235 (60.6) vs. 153 (39.4) 1541 (60.5) vs. 1008 (39.5) | 1.019 (0.937, 1.109) 1.0 (ref) | 513 3263 | 321 (62.6) vs. 193 (37.6)  2052 (62.9) vs. 1210 (37.1) | 0.959 (0.911, 1.009) 1.0 (ref) |
| **Smoking Status**  Nonsmoker   Current smoker  Past smoker | 1788 405 744 | 1051 (58.8) vs. 738 (41.3)  244 (60.2) vs. 161 (39.8)  482 (64.8) vs. 262 (35.2) | 1.0 (ref) 0.954 (0.873, 1.043) 0.999 (0.932, 1.071) | 2301 491 984 | 1418 (61.6) vs. 883 (38.4)  327 (66.6) vs. 164 (33.4)  628 (63.8) vs. 356 (36.2) | 1.0 (ref) 0.990 (0.928, 1.056) 1.020 (0.972, 1.070) |
| **AP drug use**^\|\|^  Yes   No | 1759 1178 | 1736 (98.7) vs. 23 (1.3)  40 (3.4) vs. 1138 (96.6) | NA NA | 2372 1404 | 2270 (95.7) vs. 102 (4.3)  103 (7.3) vs. 1301 (92.7) | NA NA |
| **Physician specialty**  GP/PCP/geriatrician   Cardiologist   Neurologist   Internist   Other | 114 2638 51 70 64 | 82 (71.9) vs. 32 (28.1)  1589 (60.2) vs. 1049 (39.8)  41 (80.4) vs. 10 (19.6)  36 (51.4) vs. 34 (48.6)  28 (43.8) vs. 36 (56.3) | 1.178 (1.065, 1.302) 1.0 (ref) 1.258 (1.089, 1.453) 0.843 (0.668, 1.064) 0.743 (0.557, 0.990) | 256 3277 71 82 90 | 140 (54.7) vs. 116 (45.3) 2108 (64.3) vs. 1169 (35.7)  37 (52.1) vs. 34 (47.9) 49 (59.8) vs. 33 (40.2) 39 (43.3) vs. 51 (56.7) | 0.885 (0.791, 0.991) 1.0 (ref) 0.864 (0.694, 1.077) 0.875 (0.748, 1.024) 0.783 (0.619, 0.991) |
| **Medical treatment reimbursement**  Self-pay/no coverage  Not self-pay | 274  2663 | 199 (72.6) vs. 76 (27.7)  1577 (59.2) vs. 1086 (40.8) | 1.164 (1.082, 1.252) 1.0 (ref) | 180 3596 | 118 (65.6) vs. 62 (34.4)  2255 (62.7) vs. 1341 (37.3) | 1.035 (0.977, 1.095) 1.0 (ref) |
| **Type of site**  Specialist office  Community hospital   University hospital   Other^¶^ | 911 531 1198 297 | 598 (65.6) vs. 313 (34.4)  339 (63.8) vs. 192 (36.2)  673 (56.2) vs. 525 (43.8)  166 (55.9) vs. 131 (44.1) | 1.002 (0.916, 1.097) 1.0 (ref) 0.963 (0.886, 1.045) 0.828 (0.730, 0.939) | 1019 842 1407 508 | 719 (70.6) vs. 300 (29.4) 502 (59.6) vs. 340 (40.4) 804 (57.1) vs. 603 (42.9)  348 (68.5) vs. 160 (31.5) | 1.001 (0.935, 1.073) 1.0 (ref) 0.895 (0.835, 0.960) 1.025 (0.948, 1.109) |

AF, atrial fibrillation; AP, antiplatelets; BMI, body mass index; CI, confidence interval; GP, general practitioner; LV, left ventricle; NA, not applicable; OAC, oral anticoagulant; PCP, primary care physician; ref, reference; TIA, transient ischemic attack; VKA, vitamin K antagonist.

^*^Excluding patients from Africa/the Middle East.

^†^The data of patients in combinations of oral anticoagulants treatment group are not considered. OAC use includes dabigatran, VKA, rivaroxaban, and apixaban. AP includes AP without OAC.

^‡^CIs were calculated based on the likelihood method.

^§^Prior myocardial infarction, peripheral artery disease, complex aortic plaque.

^||^AP use was defined as use at the baseline visit.

^¶^GP/primary care, outpatient healthcare centre, anticoagulation clinics, and other.
